# Systematic analysis of chromatin interactions at disease associated loci links novel candidate genes to inflammatory bowel disease

**DOI:** 10.1186/s13059-016-1100-3

**Published:** 2016-11-30

**Authors:** Claartje A. Meddens, Magdalena Harakalova, Noortje A. M. van den Dungen, Hassan Foroughi Asl, Hemme J. Hijma, Edwin P. J. G. Cuppen, Johan L. M. Björkegren, Folkert W. Asselbergs, Edward E. S. Nieuwenhuis, Michal Mokry

**Affiliations:** 1 Department of Pediatric Gastroenterology, Wilhelmina Children’s Hospital, University Medical Center Utrecht, Huispostnummer KA.03.019.0, Lundlaan 6, P.O. Box 85090, 3508 AB Utrecht, The Netherlands; 2 Department of Cardiology, Division Heart and Lungs, University Medical Center Utrecht, Utrecht, The Netherlands; 3 Vascular Biology Unit, Department of Medical Biochemistry and Biophysics, Karolinska Institutet, Stockholm, Sweden; 4 Department of Biomedical Genetics, University Medical Center Utrecht, Utrecht, The Netherlands; 5 Hubrecht Institute, Utrecht, The Netherlands; 6 Department of Genetics and Genomic Sciences, Icahn Institute for Genomics and Multiscale Biology, Icahn School of Medicine at Mount Sinai, New York, NY USA; 7 Department of Physiology, Institute of Biomedicine and Translational Medicine, University of Tartu, Tartu, Estonia; 8 Durrer Center for Cardiogenetic Research, Utrecht, The Netherlands; 9 Institute of Cardiovascular Science, University College London, London, UK

**Keywords:** Inflammatory bowel disease, Genetics, Epigenetics, Genome-wide association studies (GWAS), Enhancer elements, Chromatin interactions, DNA regulation, Candidate genes

## Abstract

**Background:**

Genome-wide association studies (GWAS) have revealed many susceptibility loci for complex genetic diseases. For most loci, the causal genes have not been identified. Currently, the identification of candidate genes is predominantly based on genes that localize close to or within identified loci. We have recently shown that 92 of the 163 inflammatory bowel disease (IBD)-loci co-localize with non-coding DNA regulatory elements (DREs). Mutations in DREs can contribute to IBD pathogenesis through dysregulation of gene expression. Consequently, genes that are regulated by these 92 DREs are to be considered as candidate genes. This study uses circular chromosome conformation capture-sequencing (4C-seq) to systematically analyze chromatin-interactions at IBD susceptibility loci that localize to regulatory DNA.

**Results:**

Using 4C-seq, we identify genomic regions that physically interact with the 92 DRE that were found at IBD susceptibility loci. Since the activity of regulatory elements is cell-type specific, 4C-seq was performed in monocytes, lymphocytes, and intestinal epithelial cells. Altogether, we identified 902 novel IBD candidate genes. These include genes specific for IBD-subtypes and many noteworthy genes including *ATG9A* and *IL10RA*. We show that expression of many novel candidate genes is genotype-dependent and that these genes are upregulated during intestinal inflammation in IBD. Furthermore, we identify *HNF4α* as a potential key upstream regulator of IBD candidate genes.

**Conclusions:**

We reveal many novel and relevant IBD candidate genes, pathways, and regulators. Our approach complements classical candidate gene identification, links novel genes to IBD and can be applied to any existing GWAS data.

**Electronic supplementary material:**

The online version of this article (doi:10.1186/s13059-016-1100-3) contains supplementary material, which is available to authorized users.

## Background

Inflammatory bowel disease (IBD) is an inflammatory disorder of the gastro intestinal tract with an intermittent, chronic, or progressive character. Studies on the pathogenesis of IBD have elucidated the involvement of a broad range of processes that mainly regulate the interaction between the intestinal mucosa, the immune system, and microbiota [1]. A role for genetics in the pathogenesis of IBD has been established through twin-based, family-based, and population-based studies [1]. Subsequently, a substantial effort to identify genetic elements involved in the IBD pathogenesis followed. In this respect, multiple genome-wide association studies (GWASs) have been performed over the past years [2–5]. In these studies, common genetic variants (single nucleotide polymorphisms (SNPs)) are assayed across the whole genome in search of variants that are significantly over-represented or under-represented in patients compared to healthy controls. Although GWASs have revealed many IBD-associated loci, for most loci the causal genes that led to the associations have not been identified. Furthermore, the majority of IBD-associated SNPs are located in non-coding DNA and therefore cannot be causal in the sense that they directly lead to amino acid changes at the protein level [2–4, 6–9]. Therefore, these SNPS are generally thought to be markers for disease-causing variants in nearby genes. This model is used in classical approaches for candidate gene identification. These approaches are mainly based on the selection of genes that have shared functional relationships and are localized in the vicinity of the identified loci [10, 11]. This has led to the identification of crucial genes and pathways involved in the IBD pathogenesis [12]. However, over the past decade it has been established that besides genes, the human genome consists of many other functional elements in the non-protein-coding regions. These regions of the genome can play a role in the pathogenesis of complex diseases. As such, many types of DNA regulatory elements (DRE), especially enhancer elements, are involved in establishing spatiotemporal gene expression patterns in a cell type-specific manner [13]. These elements are crucial in the regulation of developmental processes and in maintaining cell type-specific functionality. It is therefore now widely appreciated that part of the GWAS associations is due to sequence variation in DRE, but this information has largely been ignored in candidate gene identification [9, 14–18].

We have recently shown that 92 of 163 IBD GWAS susceptibility loci localize to DRE (identified through the presence of H3K27Ac in relevant cell types) [9]. DRE are involved in transcription regulation and establishing cell type-specific expression patterns [19]. The genes that are regulated by the IBD-associated elements are likely to play a role in IBD and can therefore be considered as IBD candidate genes. This information has not been used in previous candidate gene approaches, because the identification of these genes comes with several hurdles. Since regulatory elements can regulate genes via chromatin–chromatin interactions that comprise up to 1 Mb [20, 21], these genes cannot be identified based on their linear distance from the regulatory regions. Classical methods for candidate gene identification, that take regulatory mechanisms into account, have mainly been restricted to computational approaches [14, 16, 22, 23]. So far, a limited number of studies have shown the value of using physical interactions between regulatory elements and the genes they regulate through studying the three-dimensional (3D) nuclear conformation chromatin interactions in GWAS interpretation. These studies analyzed either single interactions (3C) or many-vs-many interactions (Hi-C) and were performed in colorectal cancer, auto-immune diseases, and multiple other diseases [24–27]. In contrast to these approaches we make use of circular chromosome conformation capture-sequencing (4C-seq), thereby increasing the number of analyzed interactions compared to 3C and increasing the resolution compared to Hi-C. Our study provides the first systematic analysis of chromatin interactions between disease-associated DRE and candidate genes in IBD. We have identified 902 novel IBD candidate genes, consisting of many noteworthy genes, for example *IL10RA*, *SMAD5*, and *ATG9A*.

## Results

### Genes interacting with DRE at IBD associated loci

**Fig. 1:**
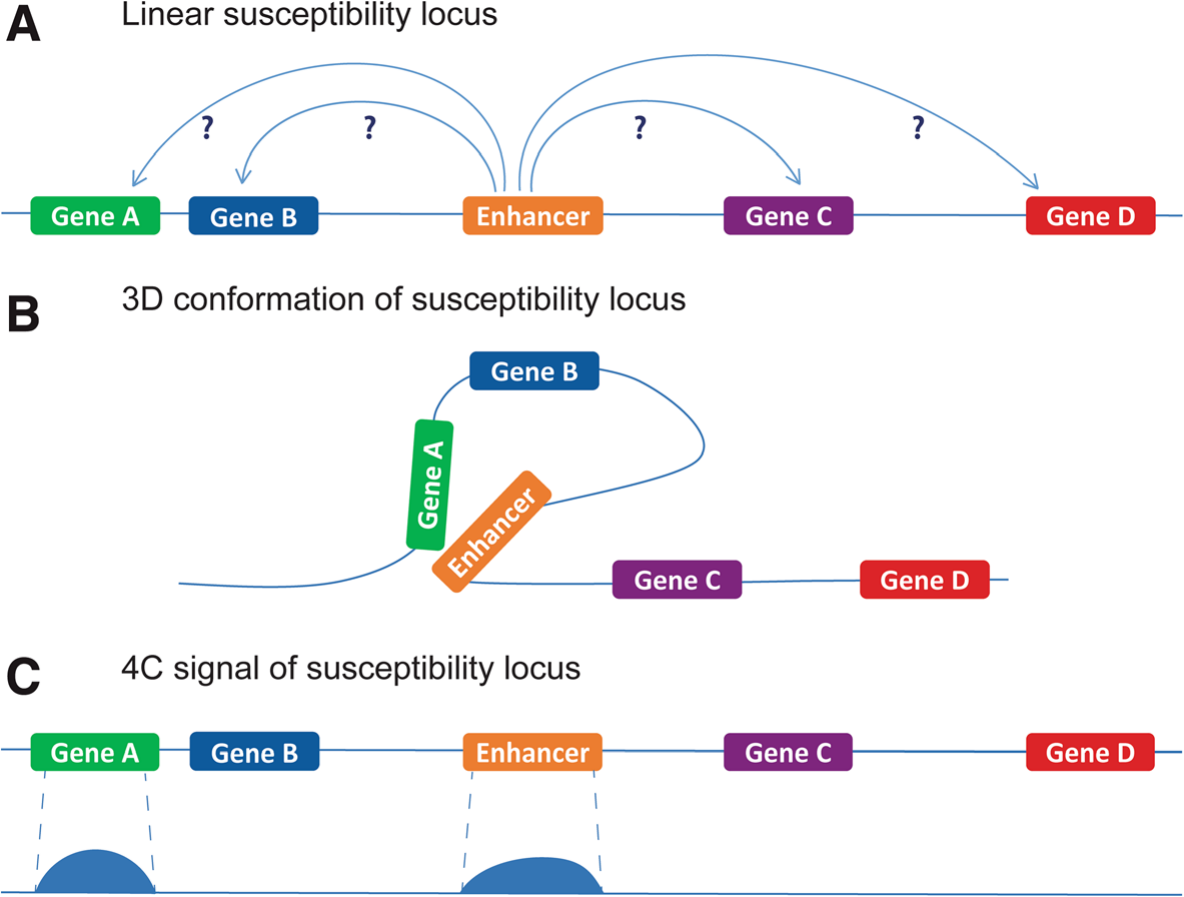
3D nuclear organization in candidate gene identification. **a** The linear organization of the genome does not provide sufficient information to predict which gene is regulated by an enhancer of interest. **b** Genes that are regulated by an enhancer form a 3D nuclear interaction. **c** The 4C-seq technique captures the 3D conformation and results in a signal around the gene that was interacting with the viewpoint (i.e. the SNP). For a detailed explanation of the 4C-seq procedure we refer to the published 4C protocol [54]. In this study, the analysis of the 3D conformation of chromatin will reveal which genes interact with an enhancer that is found at an IBD-susceptibility locus. The 4C analysis of a locus will show an interaction signal that can be mapped to the gene with which the interaction was formed. Therefore, 4C-seq can be used as a tool to use information on DNA regulation for candidate gene identification

A meta-analysis on GWASs performed in IBD resulted in the confirmation of 163 susceptibility loci [3]. We have recently shown that 92 of these 163 loci overlap with enhancer elements (regulatory elements that enhance transcription) that are active in relevant cell types for IBD (i.e. intestinal epithelial cells and immune cells) [9]. We now use this information to identify novel IBD candidate genes. We do so by identifying the genes that are regulated by these 92 regulatory elements. Since the regulated genes cannot be pinpointed by studying the linear organization of the susceptibility loci, we assayed the 3D conformation of these loci (Fig. 1). The effect of common variants, especially those in regulatory elements, is relatively mild. Therefore, it is very unlikely that a single common variant will ablate or create a whole regulatory region and its 3D interaction [28]. By the same reasoning, we do not expect that the 3D interactions in patients will be fundamentally different compared to healthy controls or cell lines. However, regulation of genes can be genotype specific [16], which demands for the identification of genes that are dysregulated in IBD. For these reasons, we decided on an experimental setup where we assay chromatin conformation in healthy control cells and a cell line, to identify genes that can be dysregulated in IBD under pathological conditions. Therefore, we have performed 92 high resolution 4C-seq experiments to cover all individual IBD susceptibility loci that overlap DRE in three cell types, thereby creating 276 individual chromatin interaction datasets. This way, we could identify all genes that physically interact with the regulatory elements that are found at IBD associated loci. As the activity of enhancers is known to be cell type-specific [19], we assayed chromatin interactions in monocytes (i.e. CD14+ fraction of PBMCs), lymphocytes (i.e. CD14- fraction of PBMCs), and in an intestinal epithelial cell line (DLD-1, derived from colorectal adenocarcinoma).

### 4C-seq identifies different sets of candidate genes in different cell types

**Fig. 2:**
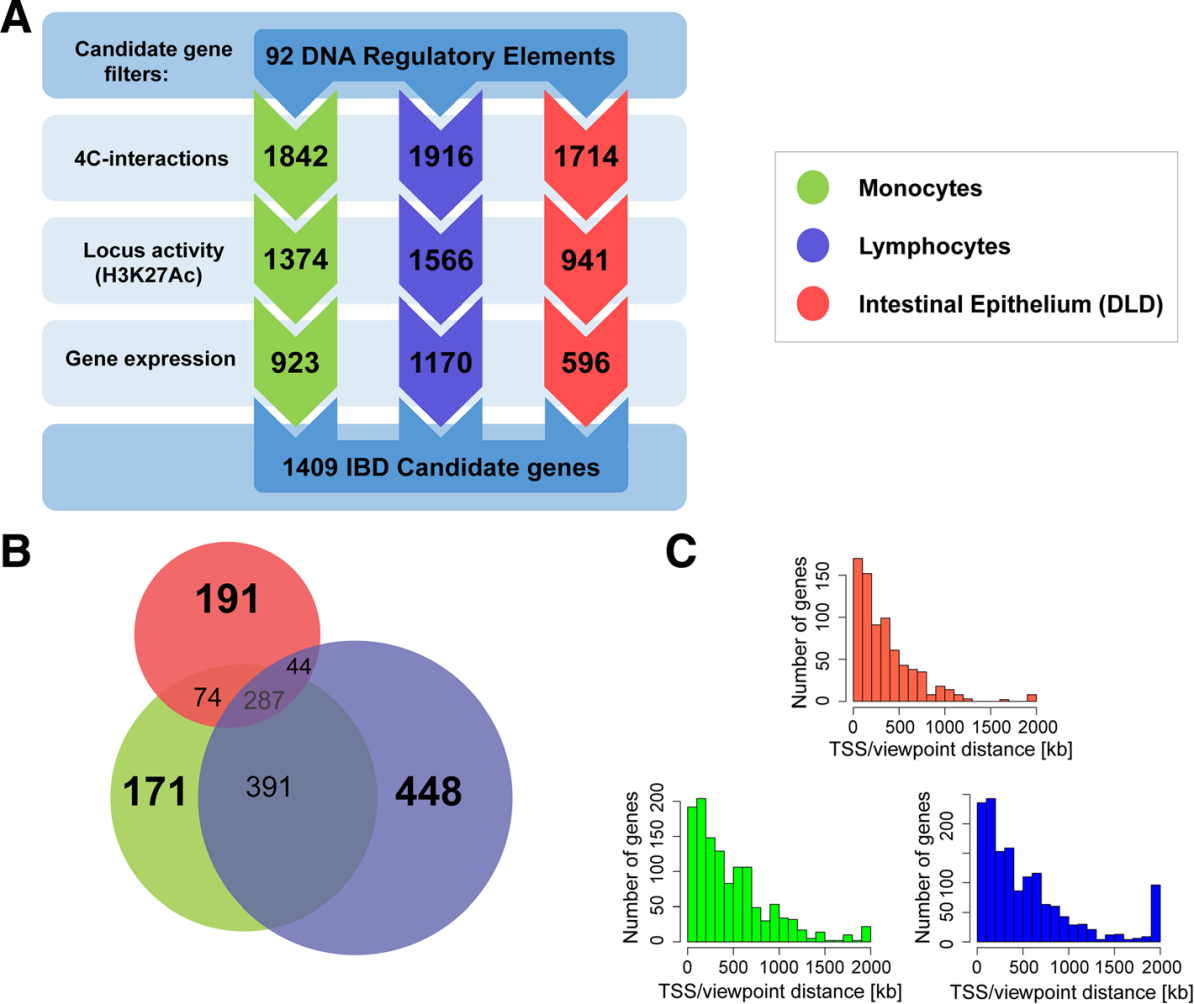
Candidate gene characteristics. **a** *Flowchart* of filtering steps that were performed to identify IBD candidate genes (4C interactions with *p* > 10^–8^; locus activity based on the co-localization of the associated variant or a variant in LD with H3K27Ac; gene expression: log_2_(RPKM) > –0.5). The number of remaining genes after each step is depicted in the corresponding *arrow*. **b** A *Venn diagram* of the candidate genes (that meet all three criteria) identified in the three separate cell types. The surface of the *circles* corresponds with the numbers of genes that are unique for one cell type and with the genes that where only two cell types overlap. The number of genes shared by all three cell types is depicted in the *center* of the *diagram*. The differences between DLDs and the immune cells is not solely due to shared active enhancers between monocytes and lymphocytes that are inactive in DLDs. To address this, Additional file 2: Figure S5 depicts a *Venn diagram* of all genes interacting with one of all (92) assayed viewpoints. These results confirm the ability of 4C-seq to detect cell type specific chromatin-chromatin interactions. **c** Distribution of the distance between the reported candidate genes and the viewpoints. The majority of the genes is located several hundreds of kilobases away from the susceptibility locus

The candidate genes that we report here all meet the following criteria: (1) the enhancer element physically interacts with the candidate gene (*p* > 10^–8^); (2) the enhancer element is active in the assayed cell type (i.e. the associated variant or a variant in LD co-localizes with the histone mark H3K27Ac) [9]; and (3) the candidate gene is expressed in the assayed cell type (log_2_(RPKM) > –0.5). With this approach we identified 1409 candidate genes: 923 genes in monocytes, 1170 in lymphocytes, and 596 in DLD-1 cells, of which 796 were shared by two or more cell types and 810 were found in only one cell type (Fig. 2a and b). We identified 902 IBD candidate genes that have not been reported by GWASs before (Table 1, Additional file 1: Table S2). Of the 92 studied loci, 22 are associated to only one of the IBD subtypes (11 to Crohn’s disease, 11 to ulcerative colitis). The candidate genes that were identified for these loci might contribute to the mechanisms that lead to the subtype-specific phenotypes. Interestingly, for two loci on chromosome 7 that give separate GWAS signals for CD (rs10486483) and UC (rs4722672), the 10 candidate genes that were identified for this CD locus were also found in the UC locus. This implies that in some cases, although the genetic risk factor is different between the subtypes, the mechanisms that underlie the genetic risk can share downstream components. Notably, this UC locus is active in intestinal epithelium, whereas the CD locus is not, which resulted in the identification of additional candidate genes for rs4722672 that are UC-specific (Table 1). Among the identified candidate genes are many noteworthy genes that have been implicated in the IBD pathogenesis, but that were never identified through GWAS associations (Table 2 [29–35]). We have now identified these novel candidate genes that have been missed by classical approaches for candidate gene identification.

**Table 1:**
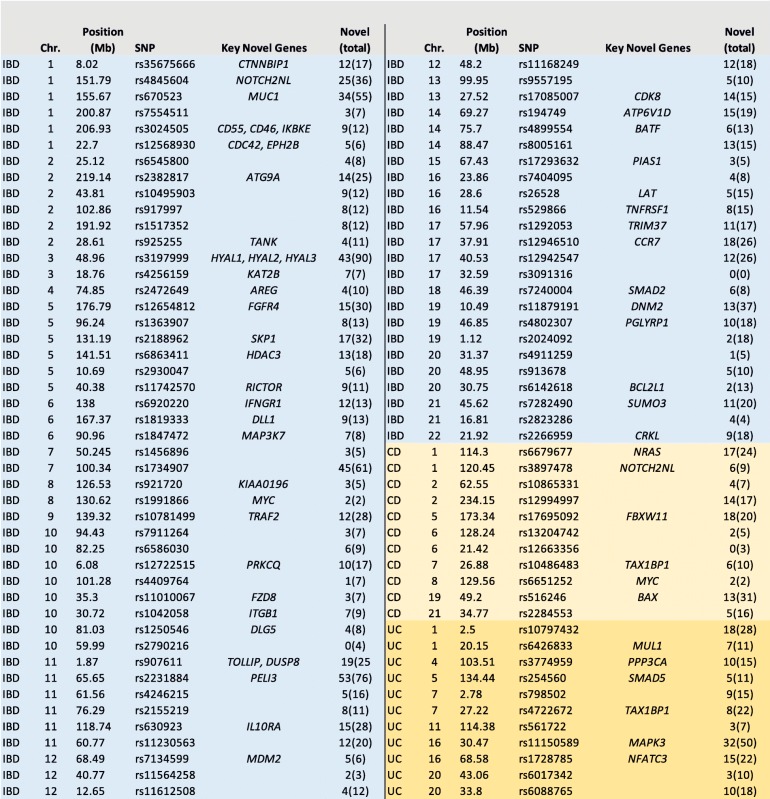
4C-seq output per locus

This table shows the 4C output for each associated IBD locus that overlaps an active regulatory element based on the presence of H3K27Ac and is arranged based on the GWAS association of the loci to either Crohn’s disease (CD), ulcerative colitis (UC), or both (IBD). The positions are given as in the GWAS in which the association was found [3] and are relative to human reference genome GRCh37. Depicted are the name of the SNP and the key novel genes that were identified at that locus. The numbers refer to the number of novel genes that were identified at that locus and, between brackets, the total number of candidate genes identified at that locus

**Table 2:** Noteworthy novel candidate genes

***ATG9A*** **:** *ATG9A* encodes autophagy related protein 9A. Autophagy plays an import role in host defense by eliminating pathogens. ATG-family member *ATG16L1* has previously been associated with Crohn’s disease [29].
***BATF*** **:** Basic leucine zipper transcription factor ATF-like (*BATF*) belongs to the activator protein 1 family that is involved in transcription regulation in all immune cells. *Batf*-deficient mice do not develop Th17 cells and do not produce IL17. Furthermore, BATF regulates cell type specific gene expression in Th2 cells, germinal center B-cells, and T-follicular helper cells [30].
***CD46/CD55*** **:** *CD46* (also known as *MCP*) and *CD55* (also known as *DAF*) are regulatory proteins expressed on surface membranes. These proteins protect the host from autologous complement-mediated injury upon activation of the complement cascade. *Daf*-deficient mice show increased epithelial damage upon induction of colitis, delayed healing, and elevated expression of proinflammatory cytokines [31].
***IL10RA*** **:** The IL10-receptor consists of the two subunits IL10RA and IL10RB. Sequence variants in genes encoding these two subunits are known to cause severe very early onset IBD in a monogenic fashion [32]. While the association of *IL10RB* with the complex form of IBD was reported by GWASs, the link with *IL10RA* was so far missing.
***SMAD5:*** SMAD5 is a downstream effector in BMP signaling. *SMAD5* expression was found to be downregulated in intestinal cells of IBD patients. Furthermore, conditional depletion of *Smad5* in mice results in increased susceptibility for development of colitis upon DSS-induction (dextran sulfate sodium) [34].

*IL10RA* interleukin 10 receptor subunit alpha, *IL10RB* interleukin 10 receptor subunit beta, *Th17 cells* T-helper 17 cells, *Th2 cells* T-helper 2 cells, *SMAD* named after their homologous genes Mothers Against Decapentaplegic (MAD) and the Small Body Size protein (SMA) in *Drosophila* and *C. Elegans*, respectively, *CD* complement-decay accelerating factor, *MCP* membrane co-factor protein, *DAF* decay accelerating factor

As expected, based on their common hematopoietic origin, the two immune cell types show larger overlap compared to DLD-1 cells (Fig. 2b, Additional file 2: Figure S5). With a median enhancer-to-gene distance of 261, 370, and 354 kbp in DLD-1, lymphocytes, and monocytes respectively, a large proportion of the genes we report are found outside the GWAS susceptibility loci (Fig. 2c). Notably, some of the interactions between IBD loci and candidate gene span over 5 Mb. For example, rs925255 shows a significant (*p* = 6.068 × 10^–9^) physical interaction with *TANK* (TRAF family member-associated NF-κB activator), a gene that is localized 30 Mb from this locus (Additional file 1: Table S2)*.*

### Validation and reproducibility of 4C-seq data

To validate the reproducibility of our data, we prepared a 4C template from lymphocytes from a different donor and performed 4C-seq for the 92 regions on this material. Additional file 2: Figure S4A shows that 91% of the candidate genes that are identified in the replicate dataset were also identified in the dataset that is used throughout this study. This demonstrates the reproducibility of the 4C technique, not only in technical, but also in biological duplicates. These results are in line with studies that have previously shown that in 3C-based methods, results from biological duplicates are highly reproducible [36]. Furthermore, we validated the reproducibility of our data by intersecting the 4C datasets with Hi-C datasets that were created in CD34+ leukocytes and a lymphoblastoid cell line [25]. This confirmed a high reproducibility by showing that 99% (CD34+) and 87% (lymphoblastoid) of the genes that were found by Hi-C were also found in our 4C data (Additional file 2: Figure S4B).

### Identified candidate genes are actively expressed

We reasoned that genes that are truly regulated by active enhancers in vivo would, on average, be more highly expressed than other genes within the region of the 4C signal. The quantitative examination of expression levels and histone modifications that mark active enhancers and promoters confirmed that the genes that were detected by our method indeed are more actively transcribed than all other genes (also than genes that were not detected by 4C and are found in the same genomic region, Additional file 2: Figures S6 and S7). These results support the detection of functional interactions by the 4C-seq approach that was executed here. Furthermore, we assessed “possible” insulator elements (i.e. insulators occupied by CTCF protein) between the 92 DRE and the candidate genes. Interestingly, the majority of interactions bypasses several CTCF sites and numerous interactions skip over 50 sites bound by CTCF (Additional file 2: Figure S8). In addition, genes that do not interact with the 4C viewpoint do not seem to have more CTCF sites between the viewpoint and their promoter compared to the interacting genes (Additional file 2: Figure S8). This is in line with observations from Hi-C datasets where 82% of long-range interactions bypass at least one CTCF site [25].

Previously, insulator regions have been shown to prevent enhancer-gene interactions [37]. We therefore investigated whether assessment of the CTCF binding can be used as an alternative to the 4C method by predicting the borders of the regions in which our candidate genes were found. We conclude that CTCF binding information cannot be used as an alternative for the 4C-based candidate gene approach presented here.

### 4C-seq candidate genes have SNP-dependent expression profiles

We hypothesize that the candidate genes that we identify are contributing to the IBD pathogenesis via impaired transcription regulation caused by variants in DRE. To test this hypothesis, we studied whether 4C-seq candidate genes show different expression profiles in different genetic backgrounds (i.e. in individuals that carry the associated SNP versus individuals that do not) through eQTL analyses [23]. We performed two different analyses in separate databases. First, we used the GTEx database [38] to test whether our approach is able to detect the eQTLs that are present in the intestinal epithelium (colon-sigmoid, colon-transverse, terminal ileum) and whole blood [38]. We performed an eQTL look-up of the 92 IBD-associated SNPs in these tissues and found 50 genes with a SNP-dependent expression profile. Interestingly, all of the 50 genes were identified by our 4C-seq approach (Additional file 3: Table S4). Second, we made use of another eQTL database (STAGE) [39] and explored the presence of candidate genes among the genes that were found to have expression levels that are dependent on the interacting SNP genotype in white blood cells. This revealed 10 candidate genes that have an eQTL in the STAGE database. Next, we analyzed all non-interacting genes within 2 Mb from the 4C viewpoint (Additional file 3: Table S4). In contrast to the interacting genes, none of the non-interacting genes showed genotype-dependent expression in the same database. These findings altogether support the capability of our method to identify the candidate genes of which the expression regulation is dependent on IBD-associated genomic variants.

### 4C-seq gene set is enriched in genes involved in inflammation in IBD patients

**Fig. 3:**
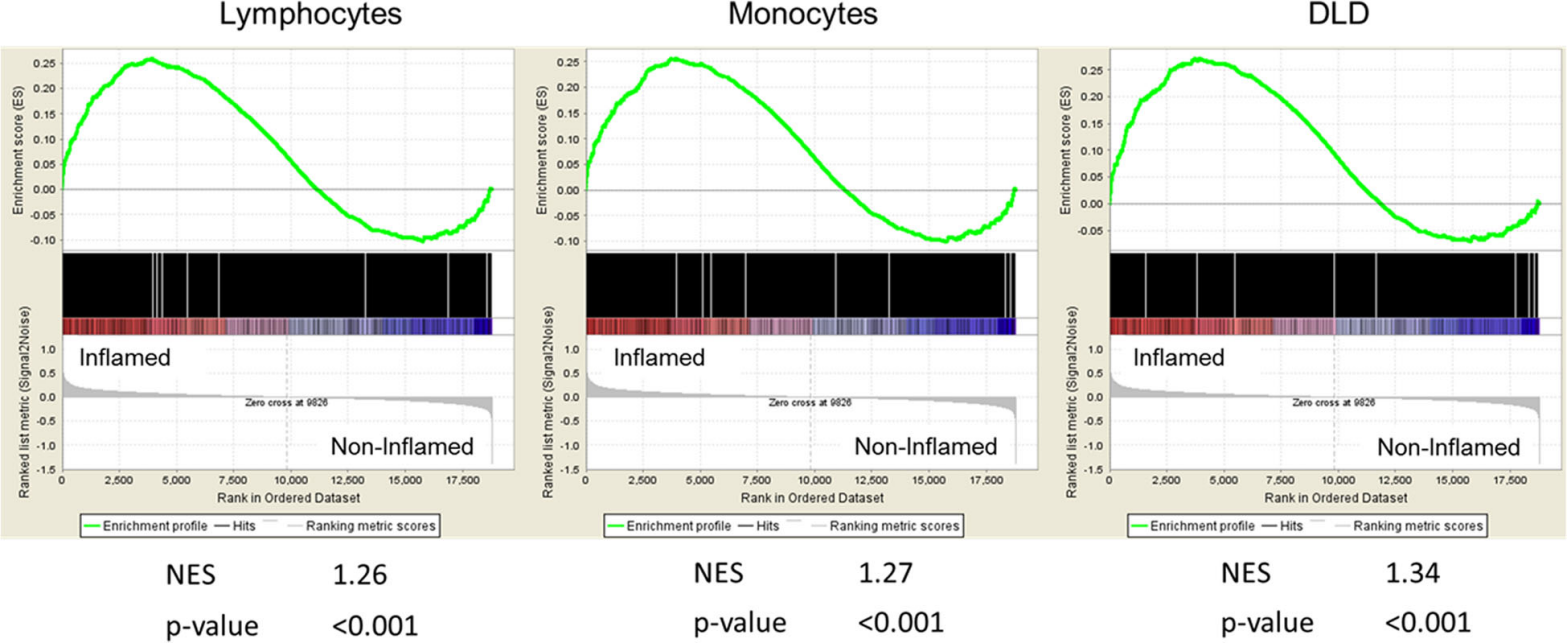
GSEA for candidate genes in intestinal inflammation in IBD. The *figure* shows the GSEA for the candidate genes reported in monocytes, lymphocytes, and DLDs. Genes that are upregulated (*red*) in inflamed compared to non-inflamed biopsies are plotted to the *left* of the *x-axes*, downregulated genes (*blue*) on the right. 4C-seq gene sets are significantly (*p* < 0.001) enriched for genes that are upregulated in the inflamed intestine of IBD patients (reflected by positive normalized enrichment score, NES). Enrichment score (ES) reflects the degree to which the 4C-seq genes sets are over-represented at the differentially expressed genes in intestinal biopsies. The nominal *p* value and the normalized enrichment score (NES, normalized for the size of the gene sets) are shown below each *graph*

After demonstrating that our method enables the identification of novel IBD candidate genes that are likely subject to SNP-dependent expression levels, we examined whether the genes we report here are involved in the major pathogenic process in IBD, namely intestinal inflammation. To address this, we performed a GSEA [40] in which we used RNA expression data of intestinal biopsies from IBD patients [41]. We compared expression levels in inflamed versus non-inflamed intestinal biopsies and tested whether the 4C-seq candidate genes were enriched among the differentially expressed genes. This analysis shows that all three 4C gene sets (monocytes, lymphocytes, and intestinal epithelium) are highly enriched (*p* < 0.001) for genes that are upregulated upon intestinal inflammation in IBD patients (Fig. 3). These results support the role of the candidate genes reported here in intestinal inflammation in IBD.

### Chromatin interactions reveal *IL10RA* and *ATG9A* as novel IBD targets

**Fig. 4:**
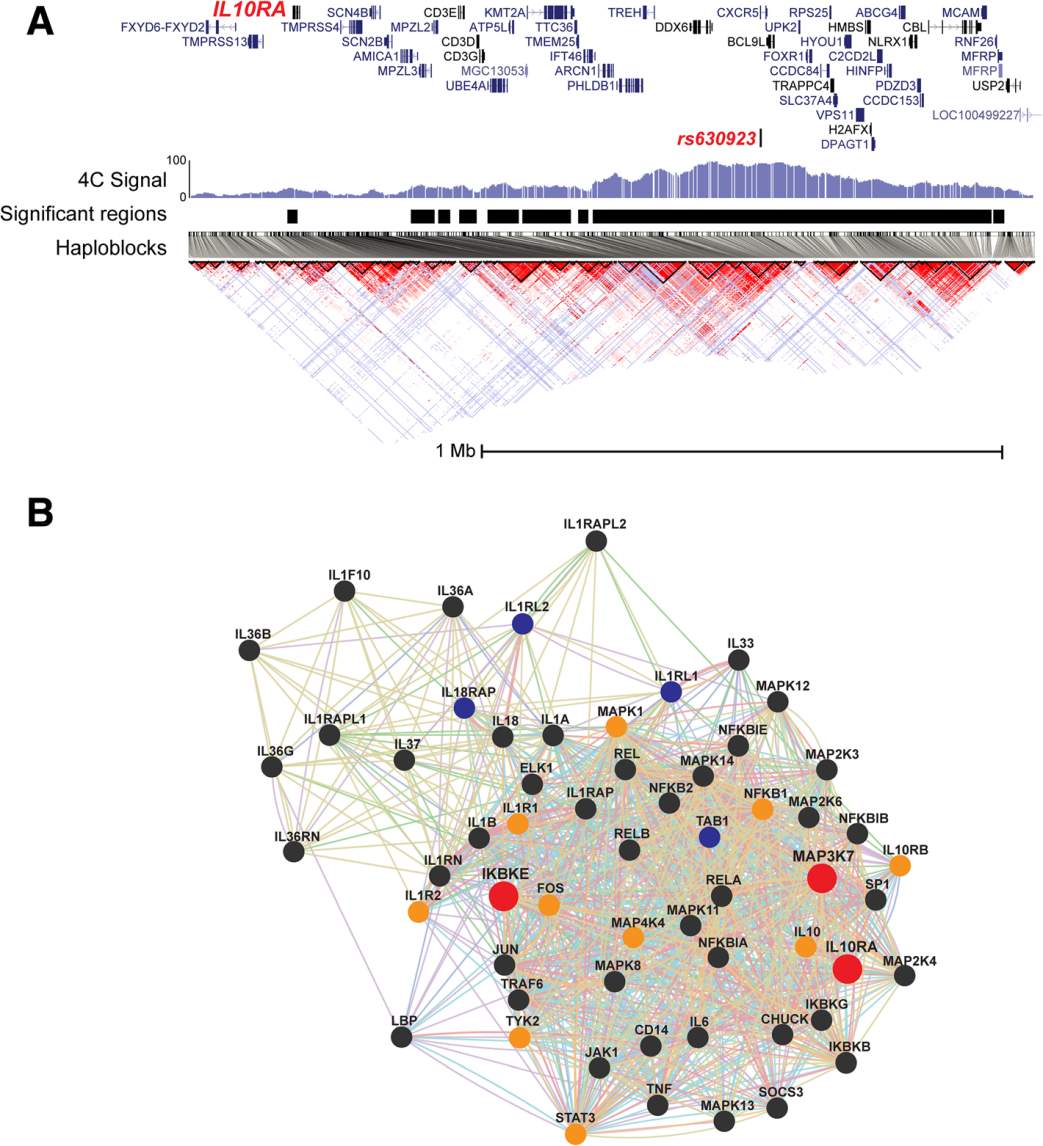
IL10RA is a novel IBD candidate gene. **a** The 4C signal from the rs630923 locus in lymphocytes; signal on the *y-axis* is depicted as the percentage of fragends covered per pixel. *Black bars* indicate significant 4C signal (*p* < 10^–8^); all coding genes located in this region are shown. The TSS of IL10RA co-localizes with a distant significant signal (~1 Mb from the viewpoint). Rs630923 and IL10RA localize to different haploblocks, meaning these regions do not co-segregate. **b** A network that consists of members of the IL10 signaling pathway. *Red dots* represent novel IBD candidate genes, *orange dots* represent candidate genes that were identified by 4C-seq as well as by GWAS, *blue dots* represent previously reported candidate genes that were not identified in the 4C-seq dataset, and *black dots* represent members of the IL10 pathway that have not been associated to IBD. Although many genes of the IL10 signaling pathway have been reported previously, we complement the network with three novel candidate genes including IL10RA

*IL10RA* is one of the newly identified candidate genes. Previously, sequence variants in genes encoding the two subunits of the interleukin 10 receptor, *IL10RA* and *IL10RB*, were found to cause severe early onset IBD in a Mendelian fashion [32]. Our 4C datasets reveal that *IL10RA* interacts with an IBD-associated enhancer element in peripheral blood lymphocytes (*p* = 4.1 × 10^–10^). Since *IL10RA* is located ~1 Mbp upstream of the associated SNP (rs630923) and is separated from the SNP by multiple haploblocks (Fig. 4a), this gene has not been identified through classical candidate gene approaches. The enhancer element that co-localizes with rs630923 is active in lymphocytes, but not in monocytes and intestinal epithelial cells (i.e. H3K27Ac marks are present only in lymphocytes). These results imply distinctive and cell type-specific regulatory pathways for *IL10RA* expression in immune cells. Besides *IL10RA*, we identified 12 candidate genes that are part of the *IL10 signaling* pathway (Fig. 4b), three of which are novel candidate genes (*IL10RA*, *IKBKE*, *MAP3K7*). These results confirm and further establish the important role of IL10 signaling in IBD.

Furthermore, we identified *ATG9A* (autophagy-related gene 9A) as a novel candidate gene, as its transcriptional start site is physically interacting with an enhancer element in the proximity of rs2382817 in DLDs and monocytes (*p* = 7.891 × 10^–13^ in monocytes, *p* = 9.787 × 10^–12^ in DLDs, Additional file 2: Figure S9). ATG9A is known to be involved in the generation of autophagosomes. Furthermore, ATG9A has been shown to dampen the innate immune response that occurs in response to microbial dsDNA. *ATG9A* knockout mice show enhanced expression of *IFN-*β, *IL6*, and *CXCL10* upon exposure to microbial dsDNA [42]. This gene is furthermore of interest to IBD, because the association of other autophagy genes to IBD is well established [6, 43, 44]. For example, patients that are homozygous for the *ATG16L* risk allele show Paneth cell granule abnormalities [45]. Based on the role *ATG9A* plays in responding to microbial dsDNA and the role *ATG16L* plays in Paneth cell degranulation, it is possible that *ATG9A* contributes to the IBD pathogenesis in monocytes and intestinal epithelial cells via distinct mechanisms.

### Pathway analysis shows cell type-specific results

**Fig. 5:**
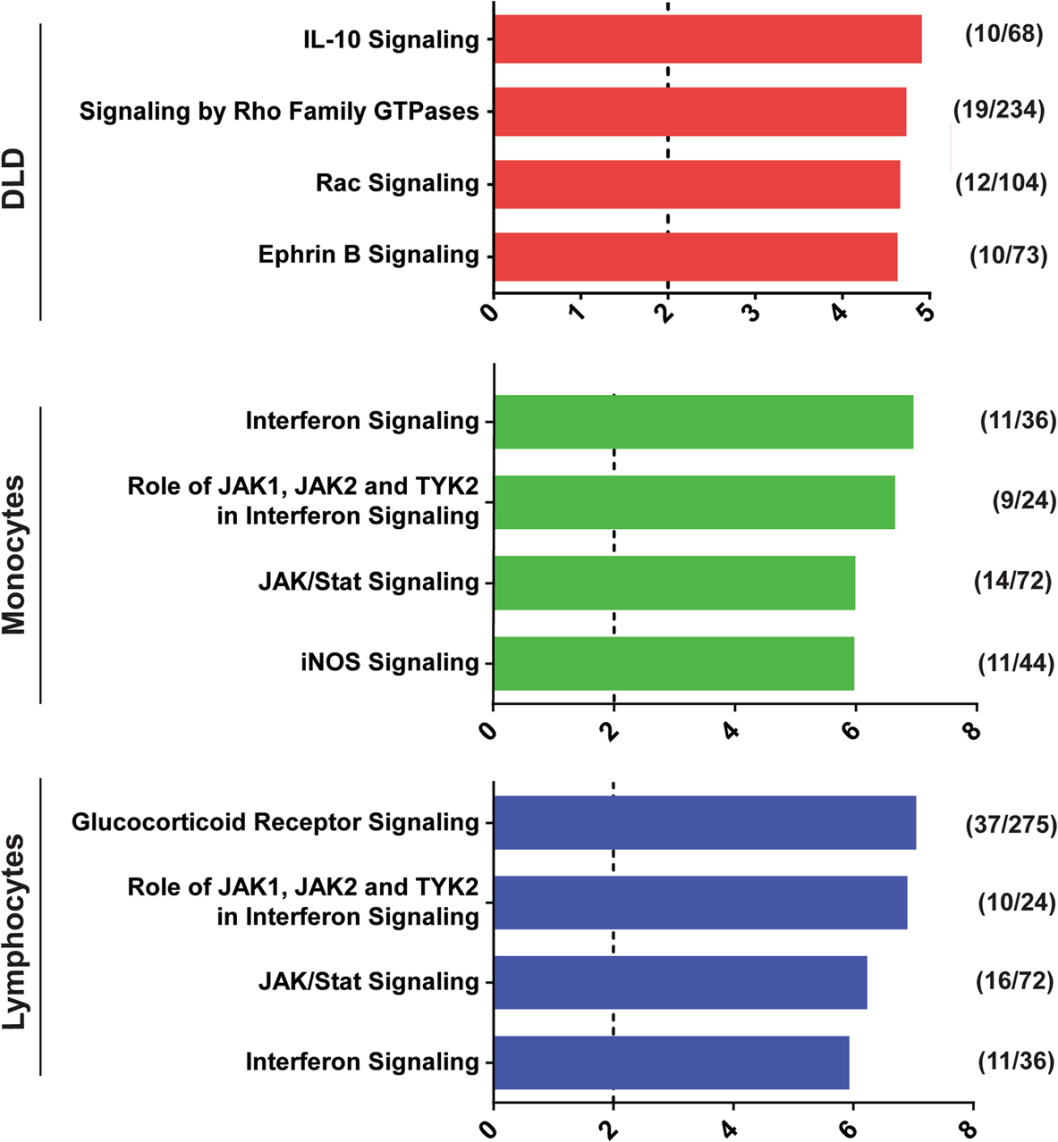
Pathways in IBD. This figure shows the pathways that are most highly enriched among the identified candidate genes in the three separate cell types. *Bars* correspond with the –Log of the *p* value, the *dashed line* indicates the threshold for significance. *Numbers* between the *brackets* show (amount of pathway members in dataset/total amount of pathway members). Pathway analyses were performed using Ingenuity Pathway Analysis (IPA, see “Methods”). All significantly enriched pathways can be found in Additional file 3: Table S4

Besides studying the individual associated loci and the genes they regulate, we aimed to elucidate the pathways in which the IBD candidate genes are involved. Since our approach enables us to determine both IBD candidate genes and the cell type in which they are likely dysregulated, we analyzed the pathogenic processes that are possibly involved in monocytes, lymphocytes, and intestinal epithelial cells. Therefore, we performed separate pathway analyses on the datasets generated in these three different cell types. This revealed that the enriched pathways in the two immune cell types are mainly similar to each other, whereas the enrichment in epithelial cells shows different pathways (Fig. 5, Additional file 4: Table S5). Notably, IL10 signaling was found to be highly enriched in the intestinal epithelium dataset. This implies that the members of this pathway are possibly dysregulated in this cell type. As this pathway is also enriched in the immune cells (Additional file 4: Table S5), it is likely that the contribution of IL10 signaling to the IBD pathogenesis can be found in the interplay between the intestinal epithelium and immune cells. Furthermore, several JAK/STAT and Interferon signaling pathways were highly enriched in both monocytes and lymphocytes. JAK-STAT is a common signaling pathway used by many cytokines. Dysregulation of the JAK-STAT pathway can lead to a plethora of immune diseases [46]. For example, tissue specific disruption of *STAT3* is known to cause an IBD-like phenotype in mice [46]. The high enrichment of many pathways that are relevant to IBD in the datasets of the separate cell types, supports the relevance of approaches that take cell type-specific role for candidate genes into account.

### Hepatocyte nuclear factor 4α (*HNF4α*) is a potential key regulator of the IBD candidate genes

**Fig. 6:**
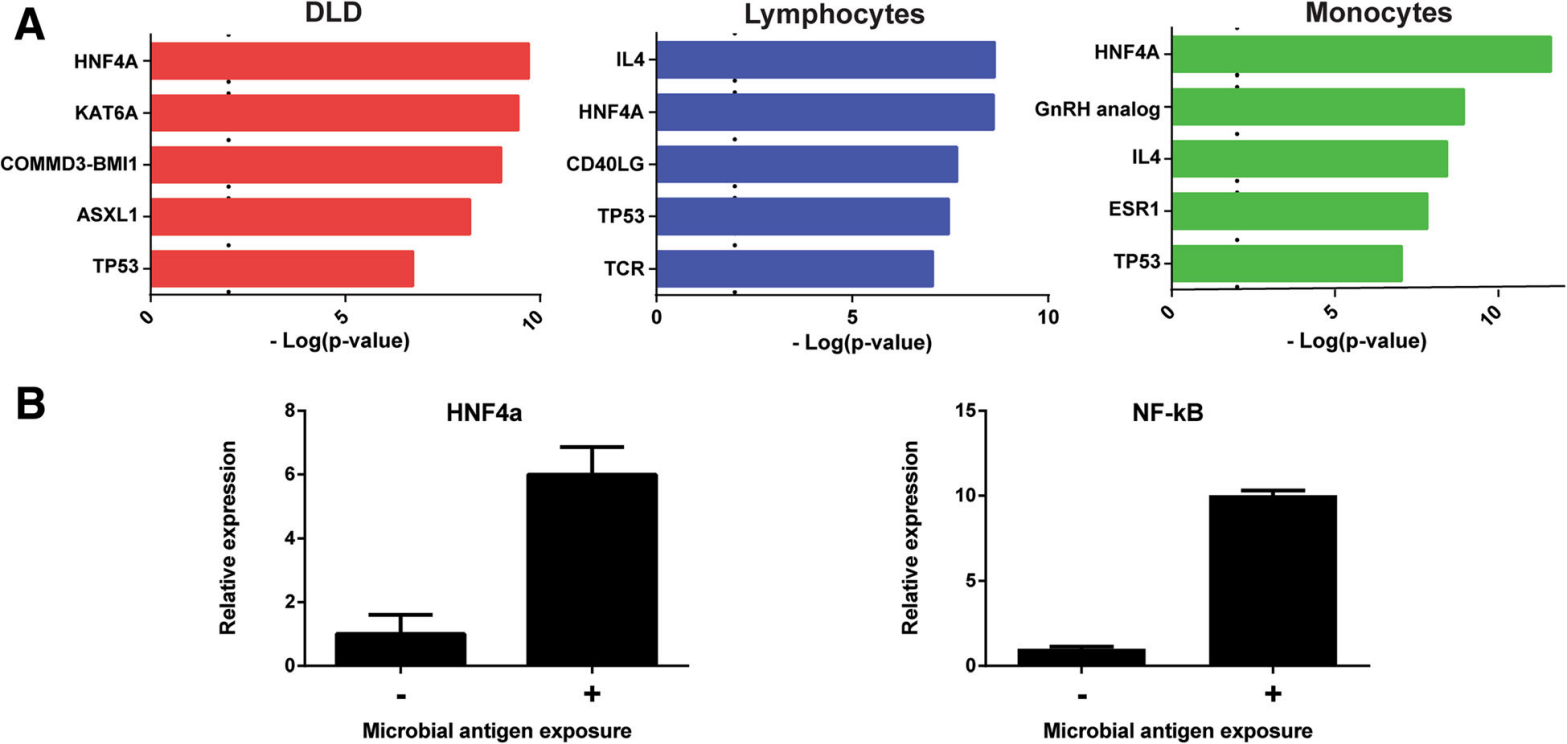
Upstream regulators of IBD candidate genes. **a** The *charts* show the upstream regulators of the identified IBD candidate genes in the separate cell types. *Bars* correspond with the –Log of the *p* value, the *dashed line* indicates the threshold for significance. The analyses were performed using Ingenuity’s Upstream Regulator Analysis (see “Methods” for more information). All significantly enriched upstream regulators can be found in Additional file 4: Table S5. **b** Relative RNA expression before (–) and upon (+) exposure of human intestinal organoids to microbial antigens. Data were normalized to β-*ACTIN* mRNA abundances. *HNF4α* and *NF-κB* are upregulated upon expose. *HNF4α* hepatocyte nuclear factor α, *KAT6A* K (lysine) acetyl transferase 6A, *COMMD3-BMI1* naturally occurring read-through transcription between the neighboring COMM domain-containing protein 3 and polycomb complex protein BMI-1, *ASXL1* additional sex combs like transcriptional regulator 1, *TP53* tumor protein p53, *IL4* Interleukin 4, *CD40LG* CD40 ligand, *TCR* T-cell receptor, *GnRN* gonadotropin releasing hormone, *ESR1* estrogen receptor 1

The 4C-seq approach reveals candidate genes based on their physical interaction with active regulatory regions. Transcription factors are important mediators in activating expression from active regulatory regions. Therefore, we aimed to determine which upstream regulators are involved in the regulation of transcriptional activity of the IBD candidate genes. We used an *in silico* analysis that determines which factors regulate expression from the candidate genes and which sets of genes that are regulated by a certain upstream regulator are enriched in our cell type-specific datasets. This analysis shows many significantly over-represented upstream regulators (Fig. 6a, Additional file 5: Table S6), including numerous transcription factors. Notably, *HNF4α* is highly enriched in all three cell types. *HNF4α* is a transcription factor that belongs to the nuclear hormone receptor superfamily [47]. Recently, the *HNF4α*-locus was associated to IBD through a GWAS [48]. Mouse studies revealed that during intestinal inflammation, HNF4α has a reduced ability to bind to active enhancers and that *Hnf4α* knock-out mice spontaneously develop colitis [49, 50].

Our study confirms that many genes that are likely dysregulated in IBD are regulated by *HNF4α*. Furthermore, *HNF4α* was found to be one of our candidate genes that was identified by a distal interaction with rs6017342 in intestinal epithelial cells (Additional file 1: Table S2). Upon exposure of intestinal organoids to bacteria lysate, we found that the epithelial response is characterized by a marked upregulation of both the *NF-κB* pathway and *HNF4α* (Fig. 6b). The kinetics of *HNF4α* expression upon epithelial responses and the enrichment of *HNF4α*-regulated genes among the IBD candidate genes propose *HNF4α* as a potential key regulator in IBD.

## Discussion

This study shows that using chromatin interactions for GWAS interpretation reveals many novel and relevant candidate genes for IBD. Specifically, we have intersected data on chromatin interactions, mRNA expression, and H3K27Ac occupation data (marking active enhancer elements) to identify IBD candidate genes. By applying 4C-seq to cell types involved in IBD, we revealed 902 novel candidate genes, consisting of multiple noteworthy genes like *SMAD5*, *IL10RA*, and *ATG9A*. Notably, many novel genes were located outside the associated loci.

There are multiple ways that can be used to identify significant interactions in 4C-seq datasets and none of these methods offer the ideal solution for all interaction ranges (long, short, inter-chromosomal), resolutions, and dynamic ranges of signal [51, 52]. In this study, we have selected a method that, to our opinion, provides a good balance between the specificity and sensitivity for interactions spanning up to several megabases. In order to reduce the amount of false-positive findings, we chose to use a stringent cutoff (*p* ≤ 10^–8^).

The identification of functional DRE–gene interactions is further established through the overlap of the candidate gene sets identified in the different cell types. Intestinal epithelial cells are developmentally and functionally very distinct from cells with a shared hematopoietic origin, in that context monocytes and lymphocytes are more alike. These differences in overlapping background are reflected by the sets of candidate genes identified in the different cell types. Specifically, lymphocytes and monocytes shared a large part of the candidate genes, whereas intestinal epithelial cells showed a more distinct set of genes (for example, monocytes share 42% and 8% of candidate genes with lymphocytes and DLD-1, respectively; Fig. 2a and Additional file 2: Figure S5). Although this approach gives a general overview of the contribution of lymphocytes to the IBD pathogenesis, it does not enable to discriminate between mechanisms in lymphocyte subsets. Analyzing a pool of cell types also decreases the sensitivity of the detection of candidate genes that are specific to a subset of cells. Therefore, in future approaches, 4C datasets for specific lymphocyte subtypes can provide more insight into the contribution of each of these cell types to the IBD pathogenesis. Furthermore, since UC is limited to the colon and CD can occur throughout the intestine, creating a 4C dataset from epithelium derived from different parts the intestine (i.e. duodenum, jejunum, ileum, and colon) might help to discriminate between the UC and CD specific pathogenic processes.

We examined the presence of eQTLs among the IBD-associated SNPs and the 4C-seq candidate genes. These analyses confirm that our approach is capable to pick up every candidate gene that was found to have SNP-dependent expression levels in tissues relevant for IBD. As expected based on the two eQTL databases that were used, not all 4C-seq candidate genes we found to have a SNP-dependent expression pattern. This is (at least in part) due to the highly context-specific nature of SNP-dependent differential expression of many eQLTs [53]. While eQTLs are usually identified at one specific cell state [53], many SNP-dependent expression patterns are only present under specific conditions (i.e. developmental stages, presence of activating stimuli, etc.), resulting in a high false-negative rate of eQTL detection. For example, many 4C-seq candidate genes might be differentially expressed between genotypes in the presence of pro-inflammatory stimuli. Our findings both confirm that our assay enables to detect genes with a SNP-dependent expression profile and underlines the need of chromatin-based techniques to identify the genes that are missed by eQTL analyses.

By using GSEA we show that the 4C-seq candidate genes are highly enriched among genes that are upregulated in inflamed intestinal biopsies from IBD patients. Since the GSEA compares inflamed versus non-inflamed intestinal tissue within patients, we cannot determine what the baseline difference in expression is between patients and healthy controls. Although the fact that a gene is upregulated upon inflammation does not show a causal relation between the (dys)regulation of that gene and the IBD phenotype, it shows the involvement of the novel 4C-seq candidate genes in IBD.

We have shown that pathway-enrichment and upstream regulator-enrichment algorithms can be used to interpret and prioritize this large candidate gene dataset. Interpretation of the 4C-seq data can be further optimized by using this data in a quantitative manner (i.e. correlating peak strength instead of using a cutoff value for peak calling). However, as with all approaches for candidate gene identification, further validation is needed to identify the causal genes for IBD. The first step towards this confirmation will in this case consist of revealing the dysregulation of the candidate gene expression upon alteration of the enhancer function in vivo.

We have profiled the chromatin interactions in primary cells from healthy controls and a cell line, to create a profile of the genes that physically interact with the IBD susceptibility loci under normal conditions in peripheral immune cells derived from healthy individuals and in an intestinal epithelium-derived cell line. As the effects of common variants in regulatory regions are relatively mild, it is improbable that a single common variant that is present in an IBD patient will ablate or create a whole regulatory region and its 3D interaction [39]. We therefore do not expect that the identification of candidate genes in cells derived from patients will reveal a substantial number of additional interactions. On the other hand, these variants are expected to cause dysregulation of the candidate genes and thereby contribute to the disease, possibly under very specific conditions, i.e. during certain stages of development or in presence of specific stimuli [16, 53].

Our study provides a proof of principle for the usage of chromatin–chromatin interactions for the identification of candidate genes. The approach presented here complements, but does not replace, previously reported approaches for candidate gene identification [11]. Candidate gene prioritization models for GWASs currently use multiple types of information, for example protein–protein interactions, expression patterns, and gene ontology. We propose that these algorithms should take chromatin interactions into account to optimize gene prioritization.

## Conclusions

We have used 4C-seq to study chromatin interactions at loci that have been associated to IBD through GWASs using 4C-seq in cell types that are involved in the pa thogenesis of IBD we identified 902 novel candidate genes, consisting of multiple noteworthy genes like *SMAD5*, *IL10RA*, and *ATG9A*.

We conclude that 4C-seq and other 3C-derived methods can be applied to candidate gene identification in diseases with a complex genetic background and complement the classical candidate gene identification approaches.

## Methods

### Cell culture

DLD-1 cells were cultured in RPMI-1640 with 10% FCS and standard supplements. Cells were harvested for 4C template preparation by trypsinization at 60–80% confluence.

### Monocyte and peripheral blood lymphocyte (PBL) isolation

Peripheral blood was collected from two healthy donors (one for monocyte isolation, one for PBL isolation) in sodium-heparin tubes. Peripheral blood mononuclear cells (PBMCs) were isolated by Ficoll-Paque gradient centrifugation. PMBCs were incubated with magnetic CD14+ microbeads (Milteny, order no. 130-050-201) according to the manufacturer’s manual. Thereafter cells were magnetically separated by the AutoMACS™ Separator; the negative fraction consisted of PBLs, the positive fraction of monocytes.

### Circular chromosome conformation capture: sequencing

#### Template preparation

For each cell type, one 4C-template was prepared. 4C-chromatin preparation, primer design, and library preparation were described previously [54]. 10 × 10^6^ cells were used for chromatin preparation per cell type (monocytes, PBLs, and DLD-1). Primer sequences are listed in Additional file 6: Table S1*.* The library preparation protocol was adapted to make it compatible with the large number of viewpoints. Details can be found in the Additional file 2: Supplementary data, Methods.

#### Sequencing

Libraries were sequenced using the HiSeq2500 platform (Illumina), producing single end reads of 50 bp.

#### Data analysis

The raw sequencing reads were de-multiplexed based on viewpoint-specific primer sequences (the datasets are accessible through GEO Series accession number GSE89441). Reads were then trimmed to 16 bases and mapped to an in silico generated library of fragends (fragment ends) neighboring all DpnII sites in human genome (NCBI37/hg19), using the custom Perl scripts. No mismatches were allowed during the mapping and the reads mapping to only one possible fragend were used for further analysis. To create the 4C signal tracks in the UCSC browser, we have generated the .*bed files with information for each mappable fragend on the coordinates and their covered/non-covered (1 or 0) status. Visualization of the tracks in the UCSC browser was done with the following settings: windowing function: mean; smoothing window: 12 pixels.

#### Identification of the interacting genes

First, we calculated the number of covered fragends within a running window of *k* fragends throughout the whole chromosome where the viewpoint is located. This binary approach (i.e. a fragend is covered or is not covered in the dataset) was chosen to overcome the influence of polymerase chain reaction (PCR)-efficiency-based biases, however this approach decreases the dynamic range of the 4C-seq and may overestimate the strength of distal interactions compared to proximal interactions. The *k* was set separately for every viewpoint so it contains on average 20 covered fragends in the area around the viewpoint (+/– 100 kbp), e.g. when 100 out of 150 fragends around the viewpoint were covered the window size was set to 30 fragends. Next, we compared the number of covered fragends in each running window to the random distribution. The windows with a significantly higher number of covered fragends compared to random distribution (*p* < 10^–8^ based on binominal cumulative distribution function; R *pbinom*) were considered as a significant 4C signal. The following criteria were defined for the identification of the candidate genes: (1) the transcriptional start site (TSS) co-localizes with a significant 4C-seq signal (*p* < 10^–8^) within 5 kbp; (2) the susceptibility variant or other variant in linkage disequilibrium (LD) co-localizes with the H3K27ac signal (that marks activating regulatory elements) in the cell type from which the 4C signal was obtained (68 loci in monocytes, 73 in lymphocytes, and 52 in intestinal epithelial cells) [9]; and (3) the gene is expressed (log2(RPKM) > –0.5) in the assayed cell type (Additional file 1: Table S2). Datasets used for expression analysis are listed in Additional file 7: Table S3. Quality measures for the 4C library preparation and sequencing can be found in Additional file 2: Supplementary data, Figures S1–S3. The use of single 4C templates per cell type was validated in a biological duplicate of the lymphocyte 4C template that is derived from a different donor (Additional file 2: Figure S4A) and the reproducibility in other chromatin interaction datasets was established by intersecting our findings with two Hi-C datasets [25] (Additional file 2: Figure S4B and Additional file 7: Table S3).

### TSS occupancy by H3K27ac and H3K4me3

The publicly available datasets of H3K27ac and H3K4me3 occupancy were accessed from the UCSC/ENCODE browser (http://genome.ucsc.edu/ENCODE/). Datasets are listed in Additional file 7: Table S3. The occupancy around 2 kbp +/– of TSS of was calculated using custom Perl scripts and Cisgenome [55] functions.

### eQTL analyses

#### GTEx

A manual look-up was performed for expression quantitative trait loci (eQTL) in the Genotype-Tissue Expression (GTEx) database (accession dates; eQTL-genes: 05-2016; *p* values: 09-2016). The presence of eQTL genes for each of the 92 IBD-associated SNPs was performed in four different tissues: colon-transverse; colon-sigmoid; small intestine-terminal ileum; and whole blood [38]. Next, for each gene for which an IBD-associated SNP turned out to be an eQTL, its presence among the 4C-seq identified genes was evaluated (Additional file 3: Table S4). All transcripts in the GTEx database that were not included in the gene annotation (UCSC genes 2009) that was used for the analysis of the 4C-seq data were removed from the analysis.

#### STAGE

eQTLs were analyzed using the Stockholm Atherosclerosis Gene Expression (STAGE) [39] dataset (Additional file 2: Supplementary data, Methods). Identified loci from GWAS for IBD were matched with imputed and genotyped SNPs and were selected for eQTL discovery. We compared the amount of eQTLs present in “SNP-candidate gene”-pairs and “SNP-control gene”-pairs. Control genes are genes within the same locus that are not interacting with the IBD-associated locus. An empirical false discovery rate was estimated for each eQTL gene by shuffling patient IDs 1000 times on genotype data as described previously [56].

### Gene set enrichment analysis (GSEA)

GSEA [40] was performed using gene expression datasets [41] from intestinal biopsies obtained from ulcerative colitis patients (datasets available at GSE11223). The “normal uninflamed sigmoid colon” and “UC inflamed sigmoid colon” were used and the fold changes in expression were calculated using the GEO2R tool [57] with default settings. Significance of the enrichment was calculated based on 1000 cycles of permutations.

### Signaling pathway analysis

The IL10 signaling pathway components were retrieved from Ingenuity Pathway Analysis (IPA®, QIAGEN Redwood City). Genes upregulated upon IL10 signaling (target genes) and genes involved in the bilirubin cascade were removed before further analysis. The interactions between the members of the IL-10 signaling pathway were visualized using the GeneMania tool <http://www.genemania.org/>.

The general pathway analysis was performed with the Ingenuity Pathway Analysis software (IPA®, QIAGEN Redwood City), based on the candidate genes from the three cell types, separately.

### Upstream regulators

Upstream regulators that are enriched regulators of the candidate genes in our datasets were identified with the Ingenuity Pathway Analysis software (IPA®, QIAGEN Redwood City), based on the candidate genes from the three cell types separately. The Ingenuity’s Upstream Regulator Analysis algorithm predicts upstream regulators from gene datasets based on the literature and compiled in the Ingenuity knowledge base.

### CTCF tracks

CTCF tracks were accessed from the UCSC/ENCODE browser (http://genome.ucsc.edu/ENCODE/). Datasets are listed in Additional file 7: Table S3.

### Tracks used for *rs630923* and *rs2382817*

All tracks were accessed from the UCSC/ENCODE browser (http://genome.ucsc.edu/ENCODE/). Datasets are listed in Additional file 7: Table S3. Haploblock structures were visualized with Haploview [58]; pairwise LD statistics of variants with a distance up to 500 kbp were used in the analyses (Fig. 4, Additional file 2: Supplementary data, Figure S9).

### Organoid culture

Colon biopsies were obtained by colonoscopy. The biopsies were macroscopically and pathologically normal. Crypt isolation and culture of human intestinal cells from biopsies have been described previously [59, 60]. In summary, human organoids were cultured in expansion medium (EM) containing RSPO1, noggin, EGF, A83-01, nicotinamide, SB202190, and WNT3A. The medium was changed every 2–3 days and organoids were passaged 1:4 every 9 days.

Five to seven days after passaging, the organoids were exposed to 10 μL sterilized *E. Coli*-lysate (control organoids were not stimulated). After 6 h of exposure, the organoids were harvested and RNA was extracted using TRIzol LS (Ambion™). Complementary DNA was synthesized by performing reverse-transcription (iScript, Biorad). Messenger RNA (mRNA) abundances were determined by real-time PCR using primer pairs that target *HNF4α and NFKB1* (Additional file 6: Table S1) with the SYBR Green method (Bio-Rad). *ACTIN* mRNA abundance was used to normalize the data.

## Additional files

Additional file 1: Table S2. 4C datasets. (XLSX 43677 kb)
Additional file 2: Supplementary data; **Figures S1–S9** and Supplementary Methods. (PDF 2295 kb)
Additional file 3: Table S4. eQTL analyses. (XLSX 16 kb)
Additional file 4: Table S5. Pathway analyses. (XLSX 65 kb)
Additional file 5: Table S6. Upstream regulators. (XLSX 135 kb)
Additional file 6: Table S1. Primer sequences. (XLSX 17 kb)
Additional file 7: Table S3. Publicly available datasets. (XLSX 11 kb)

## Abbreviations

3D: three-dimensional
4C-seq: circular chromatin conformation capture - sequencing
ATG9A: autophagy related 9A
BP: base pairs
CD: complement-decay accelerating factor
CTCF: CCCTC-binding factor
DAF: decay accelerating factor
DLD-1 cells: D.L. Dexter-1 cells
DRE: DNA regulatory element
E. Coli: *Escherichia Coli*
EQTL: expression quantitative trait loci
FCS: fetal calf serum
GWAS: genome-wide association study
H3K27Ac: acetylation of histone H3 at lysine 27
H3K4me3: trimethylation of histone H3 at lysine 4
HNF4α: hepatocyte nuclear factor 4 alpha
IKBKE: inhibitor of nuclear factor kappa-B kinase subunit epsilon
IL10: Interleukin 10
IL10RA: Interleukin 10 receptor subunit alpha
IL10RB: Interleukin 10 receptor subunit beta
JAK: Janus kinase
Kbp: kilo base pairs
LD: linkage disequilibrium
LMPCs: lamina propria mononuclear cells
MAP3K7: mitogen-activated protein kinase kinase kinase 7
Mbp: mega base pairs
MCP: membrane co-factor protein
NFKB: nuclear factor kappa B
PBL: peripheral blood lymphocytes
PBMC: peripheral blood mononuclear cells
PCR: polymerase chain reaction
PIAS1: protein inhibitor of activated STAT 1
RPKM: reads per kilobase of exon per million reads mapped
RPMI medium: Roswell Park Memorial Institute medium
SMAD: named after their homologous genes Mothers Against Decapentaplegic (MAD) and the Small Body Size protein (SMA) in Drosophila and C. Elegans, respectively
SNP: single nucleotide polymorphism
STAT: signal transducer and activator of transcription
TANK: TRAF family member-associated NFKB activator
TGFβ-1: transforming growth factor beta-1
Th17 cells: T-helper 17 cells
Th2 cells: T-helper 2 cells
TNF: tumor necrosis factor
TSS: transcriptional start site
UCSC: University of California, Santa Cruz

## References

[1] Kaser A, Zeissig S, Blumberg RS (2010). Inflammatory bowel disease. Annu Rev Immunol..

[2] Franke A, McGovern DP, Barrett JC, Wang K, Radford-Smith GL, Ahmad T (2010). Genome-wide meta-analysis increases to 71 the number of confirmed Crohn’s disease susceptibility loci. Nat Genet..

[3] Jostins L, Ripke S, Weersma RK, Duerr RH, McGovern DP, Hui KY (2012). Host-microbe interactions have shaped the genetic architecture of inflammatory bowel disease. Nature..

[4] Anderson CA, Boucher G, Lees CW, Franke A, D’Amato M, Taylor KD (2011). Meta-analysis identifies 29 additional ulcerative colitis risk loci, increasing the number of confirmed associations to 47. Nat Genet..

[5] Liu JZ, van Sommeren S, Huang H, Ng SC, Alberts R, Takahashi A (2015). Association analyses identify 38 susceptibility loci for inflammatory bowel disease and highlight shared genetic risk across populations. Nat Genet..

[6] Rioux JD, Xavier JR, Taylor KD, Silverberg MS, Goyette P, Huett A (2007). Genome-wide association study identifies new susceptibility loci for Crohn disease and implicates autophagy in disease pathogenesis. Nat Genet..

[7] Libioulle C, Louis E, Hansoul S, Sandor C, Farnir F, Franchimont D (2007). Novel Crohn disease locus identified by genome-wide association maps to a gene desert on 5p13.1 and modulates expression of PTGER4. PLoS Genet.

[8] Duerr RH, Taylor KD, Brant SR, Rioux JD, Silverberg MS, Daly MJ (2006). A genome-wide association study identifies IL23R as an inflammatory bowel disease gene. Science..

[9] Mokry M, Middendorp S, Wiegerinck CL, Witte M, Teunissen H, Meddens CA (2014). Many inflammatory bowel disease risk loci include regions that regulate gene expression in immune cells and the intestinal epithelium. Gastroenterology..

[10] Raychaudhuri S, Plenge RM, Rossin EJ, Ng AC, Purcell SM, International Schizophrenia Consortium (2009). Identifying relationships among genomic disease regions: predicting genes at pathogenic SNP associations and rare deletions. PLoS Genet.

[11] Wang K, Li M, Bucan M (2007). Pathway-based approaches for analysis of genomewide association studies. Am J Hum Genet..

[12] Rivas MA, Beaudoin M, Gardet A, Stevens C, Sharma Y, Zhang CK (2011). Deep resequencing of GWAS loci identifies independent rare variants associated with inflammatory bowel disease. Nat Genet..

[13] Shlyueva D, Stampfel G, Stark A (2014). Transcriptional enhancers: from properties to genome-wide predictions. Nat Rev Genet..

[14] Maurano MT, Humbert R, Rynes E, Thurman RE, Haugen E, Wang H (2012). Systematic localization of common disease-associate variation in regulatory DNA. Science.

[15] Kleinjan DJ, Coutinho P (2009). Cis-ruption mechanisms: Disruption of cis-regulatory control as a cause of human genetic disease. Brief Funct Genomic Proteomic..

[16] Schaub MA, Boyle AP, Kundaje A, Frazer KA (2012). Linking disease associations with regulatory information in the human genome Toward mapping the biology of the genome. Genome Res.

[17] McVicker G, van de Geijn B, Degner JF, Cain CE, Banovich NE, Raj A (2013). Identification of genetic variants that affect histone modifications in human cells. Science..

[18] Kilpinen H, Waszak SM, Gschwind AR, Raghav SK, Witwicki RM, Orioli A (2013). Coordinated effects of sequence variation on DNA binding, chromatin structure, and transcription. Science..

[19] Heintzman ND, Hon GC, Hawkins RD, Kheradpour P, Stark A, Harp LF (2009). Histone modifications at human enhancers reflect global cell-type-specific gene expression. Nature..

[20] de Laat W, Klous P, Kooren J, Noordermeer D, Palstra RJ, Simonis M (2008). Three-dimensional organization of gene expression in erythroid cells. Curr Top Dev Biol..

[21] Hughes JR, Roberts N, McGowan S, Hay D, Giannoulatou E, Lynch M (2014). Analysis of hundreds of cis-regulatory landscapes at high resolution in a single, high-throughput experiment. Nat Genet..

[22] Thurman RE, Rynes E, Humbert R, Vierstra J, Maurano MT, Haugen E (2012). The accessible chromatin landscape of the human genome. Nature..

[23] Nica AC, Dermitzakis ET (2013). Expression quantitative trait loci: present and future. Philos Trans R Soc Lond Ser B Biol Sci..

[24] Wright JB, Brown SJ, Cole MD (2010). Upregulation of c-MYC in cis through a large chromatin loop linked to a cancer risk-associated single-nucleotide polymorphism in colorectal cancer cells. Mol Cell Biol.

[25] Mifsud B, Tavares-Cadete F, Young AN, Sugar R, Schoenfelder S, Ferreira L (2015). Sup Mapping long-range promoter contacts in human cells with high-resolution capture Hi-C. Nat Genet..

[26] Jäger R, Migliorini G, Henrion M, Kandaswamy R, Speedy HE, Heindl A (2015). Capture Hi-C identifies the chromatin interactome of colorectal cancer risk loci. Nat Commun..

[27] Martin P, McGovern A, Orozco G, Duffus K, Yarwood A, Schoenfelder S (2015). Capture Hi-C reveals novel candidate genes and complex long-range interactions with related autoimmune risk loci. Nat Commun..

[28] Sladek FM, Zhong W, Lai E, Darnell JE (1990). Liver-enriched transcription factor HNF-4 is a novel member of the steroid hormone receptor superfamily. Genes Dev..

[29] Barrett JC, Lee JC, Lees CW, Prescott NJ, Anderson CA, UK IBD Genetics Consortium (2009). Genome-wide association study of ulcerative colitis identifies three new susceptibility loci, including the HNF4A region. Nat Genet.

[30] Dekkers JF, Wiegerinck CL, de Jonge HR, Bronsveld I, Janssens HM, de Winter-de Groot KM (2013). A functional CFTR assay using primary cystic fibrosis intestinal organoids. Nat Med..

[31] Vernot B, Stergachis AB, Maurano MT, Viestra J, Neph S, Thurman RE (2012). Personal and population genomics of human regulatory variation. Genome Res..

[32] Chahar S, Gandhi V, Yu S, Desai K, Cowper-Sal-Iari R, Kim Y (2014). Chromatin profiling reveals regulatory network shifts and a protective role for hepatocyte nuclear factor 4α during colitis. Mol Cell Biol..

[33] Saitoh T, Akira S (2010). Regulation of innate immune responses by autophagy-related proteins. J Cell Biol..

[34] Murphy TL, Tussiwand R, Murphy KM (2013). Specificity through cooperation: BATF-IRF interactions control immune-regulatory networks. Nat Rev Immunol..

[35] Darsigny M, Babeu JP, Dupuis AA, Furth EE, Seidman EG, Levy E (2009). Loss of hepatocyte-nuclear-factor-4alpha affects colonic ion transport and causes chronic inflammation resembling inflammatory bowel disease in mice. PLoS One..

[36] de Wit E, Vos ES, Holwerda SJ, Valdes-Quezada C, Verstegen MJ, Teunissen H (2015). CTCF binding polarity determines chromatin looping. Mol Cell..

[37] Raviram R, Rocha PP, Muller CL, Miraldi ER, Badri S, Fu Y (2016). 4C-ker: a method to reproducibly identify genome-wide interactions captured by 4C-Seq experiments. PLoS Comput Biol..

[38] Fairfax BP, Humburg P, Makino S, Naranbhai V, Wong D, Lau E (2014). Innate immune activity conditions the effect of regulatory variants upon monocyte gene expression. Science..

[39] van de Werken HJG, de Vree PJ, Splinter JE, Holwerda SJ, Klous P, de Wit E (2012). 4C technology: protocols and data analysis. Methods Enzymol..

[40] Ji H, Jiang H, Ma W, Johnson DS, Myers RM, Wong WH (2008). An integrated software system for analyzing ChIP-chip and ChIP-seq data. Nat Biotechnol..

[41] GTEx Consortium (2013). The Genotype-Tissue Expression (GTEx) project. Nat Genet.

[42] Hägg S, Skogsberg J, Lundstrom J, Noori P, Nilsson R, Zhong H (2009). Multi-organ expression profiling uncovers a gene module in coronary artery disease involving transendothelial migration of leukocytes and LIM domain binding 2: the Stockholm Atherosclerosis Gene Expression (STAGE) study. PLoS Genet..

[43] Foroughi Asl H, Talukdar HA, Kindt AS, Jain RK, Ermel R, Ruusalepp A (2015). Expression quantitative trait Loci acting across multiple tissues are enriched in inherited risk for coronary artery disease. Circ Cardiovasc Genet..

[44] Subramanian A, Tamayo P, Mootha VK, Mukherjee S, Elbert BL, Gillette MA (2005). Gene set enrichment analysis: a knowledge-based approach for interpreting genome-wide expression profiles. Proc Natl Acad Sci U S A..

[45] Noble CL, Abbas AR, Cornelius J, Lees CW, Ho GT, Toy K (2008). Regional variation in gene expression in the healthy colon is dysregulated in ulcerative colitis. Gut..

[46] NCBI. GEO2R. http://www.ncbi.nlm.nih.gov/geo/geo2r/.

[47] Barrett JC, Fry B, Maller J, Daly MJ (2005). Haploview: analysis and visualization of LD and haplotype maps. Bioinformatics..

[48] Sato T, Stange DE, Ferrante M, Vries RG, Van Es JH, Van den Brink S (2011). Long-term expansion of epithelial organoids from human colon, adenoma, adenocarcinoma, and Barrett’s epithelium. Gastroenterology..

[49] Lin F, Spencer D, Hatala DA, Levine AD, Medof ME (2004). Decay-accelerating factor deficiency increases susceptibility to dextran sulfate sodium-induced colitis: role for complement in inflammatory bowel disease. J Immunol..

[50] Glocker EO, Kotlarz D, Boztug K, Gertz EM, Schaffer AA, Noyan F (2009). Inflammatory bowel disease and mutations affecting the interleukin-10 receptor. N Engl J Med..

[51] Liu B, Tahk S, Yee KM, Fan G, Shuai K (2010). The ligase PIAS1 restricts natural regulatory T cell differentiation by epigenetic repression. Science..

[52] Allaire JM, Darsigny M, Marcoux SS, Roy SA, Schmouth JF, Umans L (2011). Loss of Smad5 leads to the disassembly of the apical junctional complex and increased susceptibility to experimental colitis. Am J Physiol Gastrointest Liver Physiol..

[53] Portillo JC, Greene A, Schwartz I, Subauste MC, Subauste CS (2015). Blockade of CD40-TRAF2,3 or CD40-TRAF6 is sufficient to inhibit pro-inflammatory responses in non-haematopoietic cells. Immunology.

[54] Sanyal A, Lajoie BR, Jain G, Dekker J (2012). The long-range interaction landscape of gene promoters. Nature..

[55] Bell AC, West AG, Felsenfeld G (1999). The protein CTCF is required for the enhancer blocking activity of vertebrate insulators. Cell..

[56] Saitoh T, Fujita N, Hayashi T, Takahara K, Satoh T, Lee H (2009). Atg9a controls dsDNA-driven dynamic translocation of STING and the innate immune response. Proc Natl Acad Sci U S A..

[57] Hampe J, Franke A, Rosenstiel P, Till A, Teuber M, Huse K (2007). A genome-wide association scan of nonsynonymous SNPs identifies a susceptibility variant for Crohn disease in ATG16L1. Nat Genet..

[58] Parkes M, Barrett JC, Prescott NJ, Tremelling M, Anderson CA, Fisher SA (2007). Sequence variants in the autophagy gene IRGM and multiple other replicating loci contribute to Crohn’s disease susceptibility. Nat Genet..

[59] Cadwell K, Liu JY, Brown SL, Miyoshi H, Loh J, Lennerz JK (2008). A key role for autophagy and the autophagy gene Atg16l1 in mouse and human intestinal Paneth cells. Nature..

[60] Shuai K, Liu B (2003). Regulation of JAK-STAT signalling in the immune system. Nat Rev Immunol..

