# Supplementary material for: Systematic analysis of chromatin interactions at disease associated loci links novel candidate genes to inflammatory bowel disease
Source: Genome Biol. 2016 Nov 30;17:247. doi: 10.1186/s13059-016-1100-3 (PMC5131449; doi:10.1186/s13059-016-1100-3)
Supplement: Additional file 2: — Supplementary data; Figures S1–S9 and Supplementary Methods. (PDF 2295 kb) [file 13059_2016_1100_MOESM2_ESM.pdf]

SUPPLEMENTARY DATA

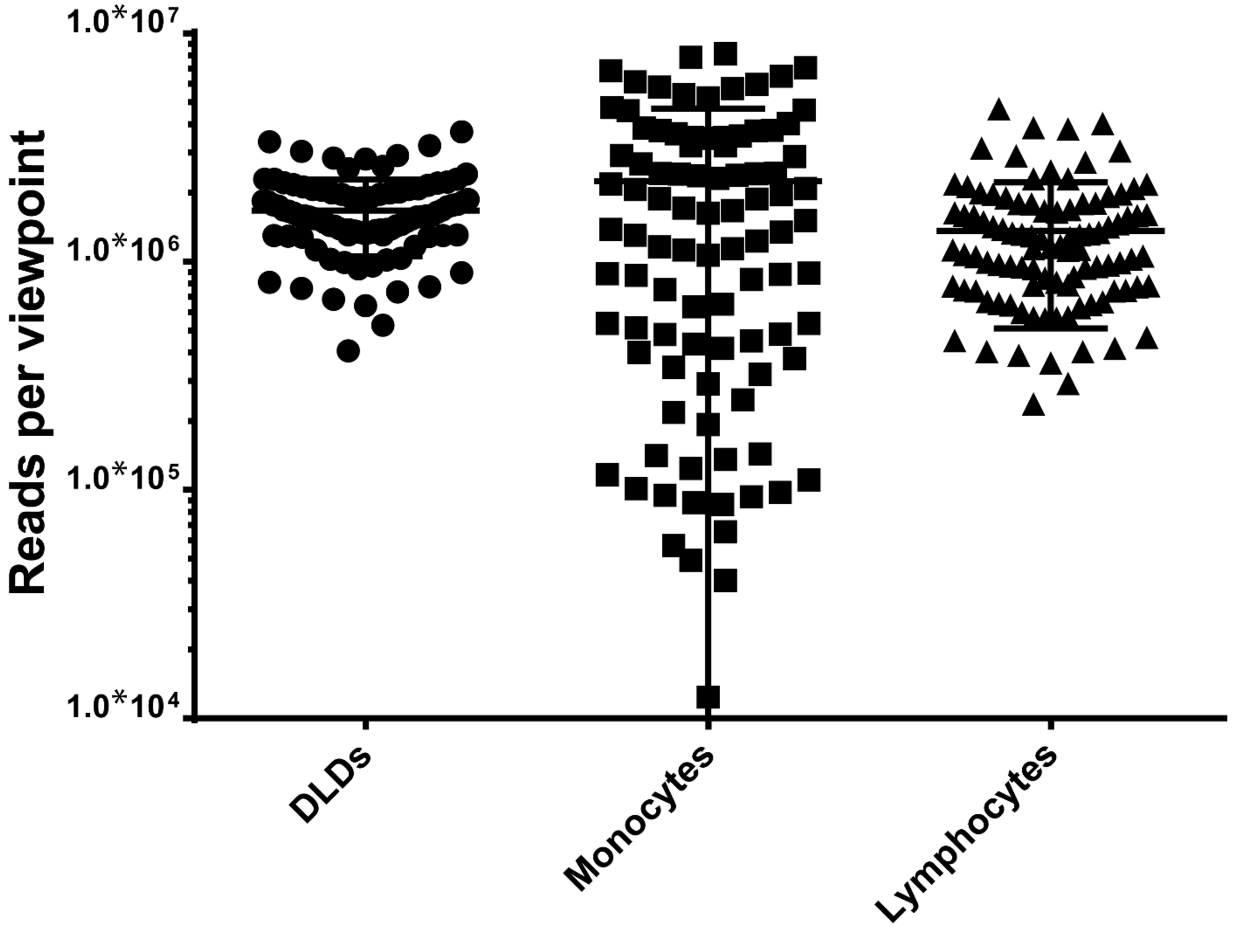

Supplementary figure 1. Number of reads per dataset. *Number of sequencing reads are shown for each of the 92 viewpoints in the three assayed cell types. Each dot represents one of the 276 viewpoints. The number of reads per dataset, although high in all cell types, is more variable in monocytes. This is due to the usage of a different pcr-multiplexing strategy in monocytes (see materials and methods for details). Datasets that consist of less than $1*10^5$ reads, do not compromise on complexity.*

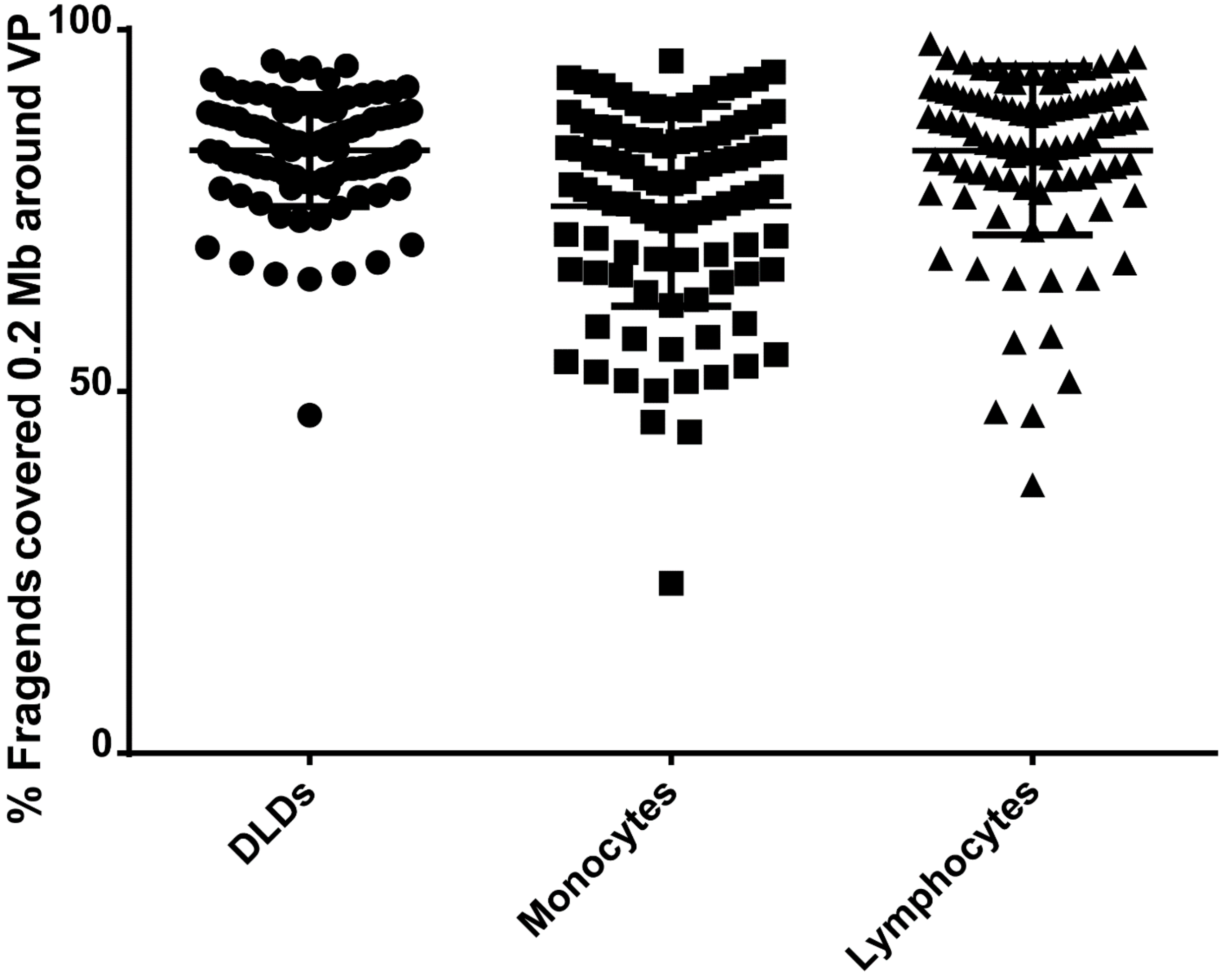

Supplementary figure 2. Percentage of fragends covered in a region of 0.2 Mb surrounding the viewpoint. *This quality measure provides an indication of the complexity of the sequenced libraries, libraries with a percentage of >40% are considered to be of high complexitiy.*[17] *Each dot represents one of the 276 viewpoints.*

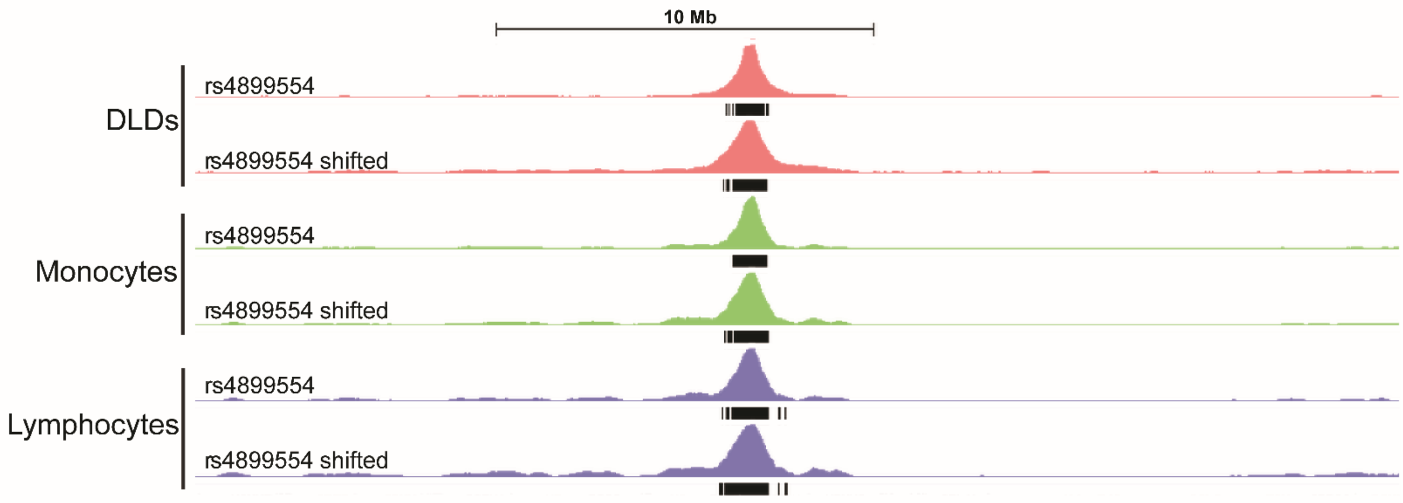

Supplementary figure 3. Different primer pairs in the same region give similar signals. *The 4C signal is shown for one viewpoint for which two primer pairs were designed (2620 bp apart). The presented primer pairs give similar signals within each cell type.*

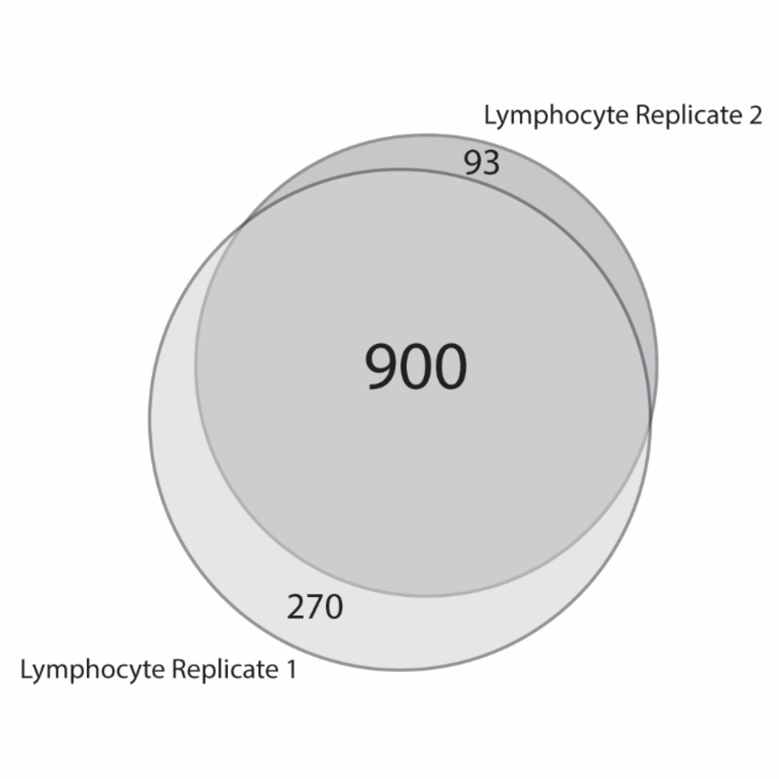

A

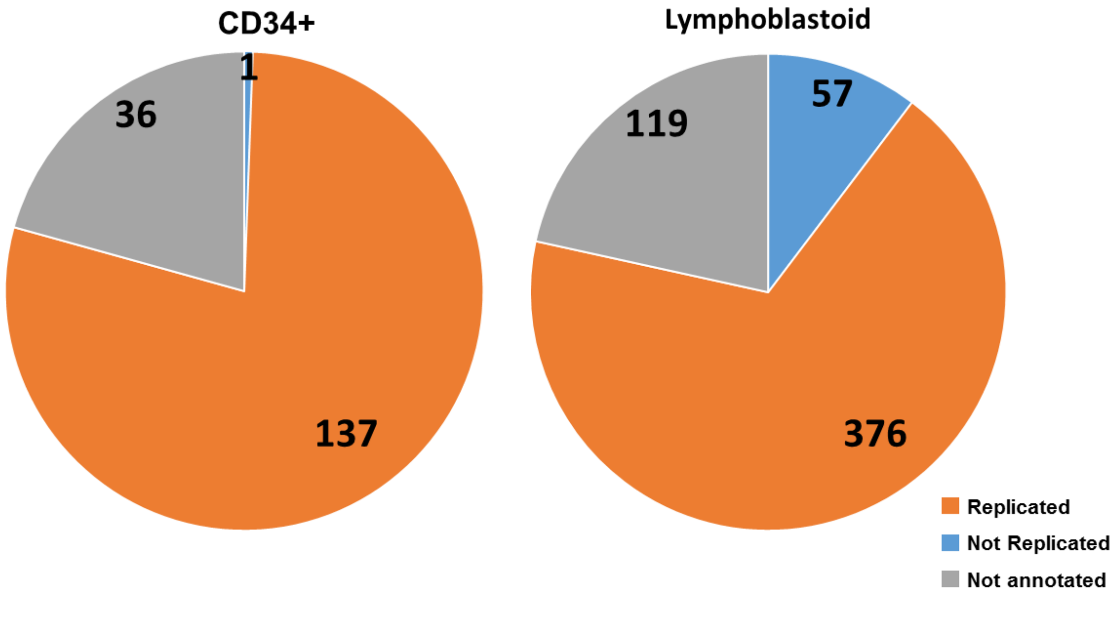

B

Supplementary figure 4. Validation dataset and overlap with Hi-C datasets. A) *Replication of the 4C-seq experiment on a 4C-template that was prepared from lymphocytes of a different donor. 91% of the candidate genes that were identified in the 2nd replicate were also identified in the dataset that is used throughout this study. This demonstrated the reproducibility of the 4C-technique; not only in technical, but also in biological duplicates. The replication dataset can be found in Supplementary table 2.* ***B)*** *To*

study the reproducibility of our results in available chromosome interaction datasets we intersected our data with two Hi-C datasets[1] that were created in CD34+ leukocytes and a lymphoblastoid cell line. First, we identified all gene promoters in the Hi-C datasets that were found to interact within 5 kB of the genomic locus of the 4C viewpoints (i.e. loci of the SNPs that we identified as being active by H3K27Ac marks in lymphocytes, Supplementary table 2), this resulted in 174 and 552 genes in the CD34+ leukocytes and lymphoblastoid cell line respectively. Next we excluded the genes that were not annotated in the same way or were missing in the annotation that was used for the 4C-seq datasets (genes in grey). Finally, we determined how many genes we were able to replicate in our study (genes in orange). This confirmed the reproducibility by showing that 99% (CD34+) and 87% (lymphoblastoid) of the genes that were found by Hi-C were also found in the 4C-data presented here (these percentages do not include the genes that were differently annotated between the Hi-C and 4C data and could therefore not be studied in this comparison).

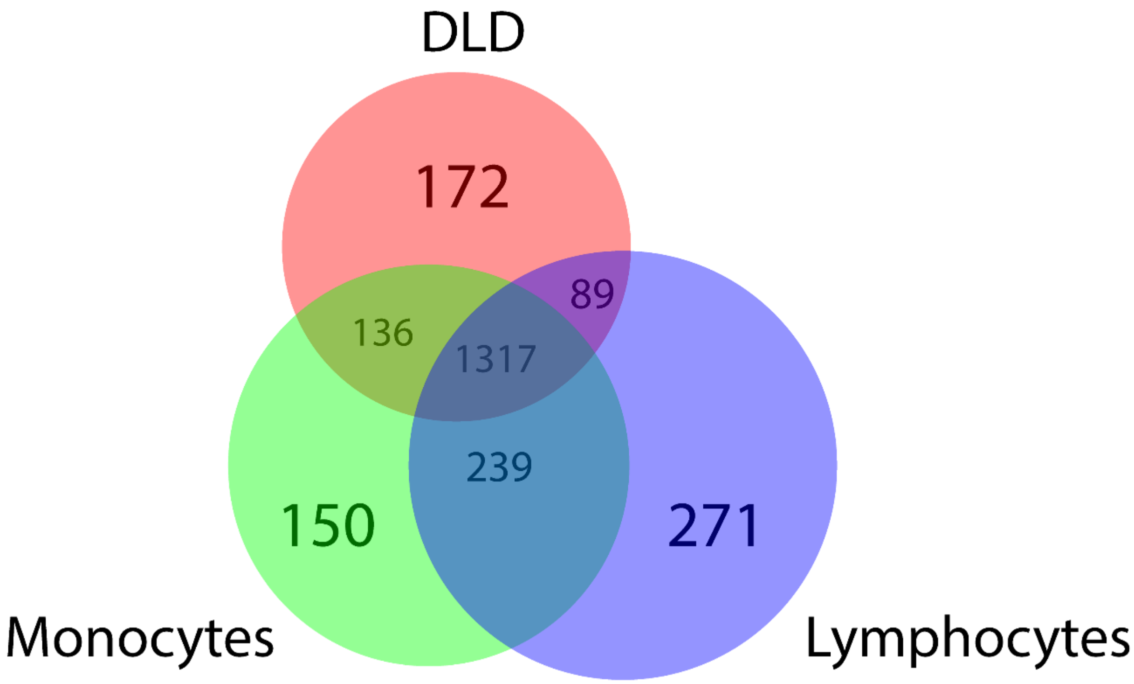

Supplementary figure 5. Overlap between interactions identified in different cell types. *This figure shows the number of genes that co-localize with a significant 4C signal in the three cell types. The surface of the circles corresponds with the genes unique for one cell type and the genes that overlap only two cell types. The number of genes shared by all three cell types is depicted in the center of the diagram. All cell types show a distinctive set of genes. As expected, monocytes and lymphocytes share more genes compared to DLD-1. In order to prevent possible biases based on cell type differences based on expression or enhancer activity, all genes that colocalize with 4C signal in any of the 92 viewpoints in any of the 3 cell types (regardless of enhancer activity of gene expression) have been used in this analysis.*

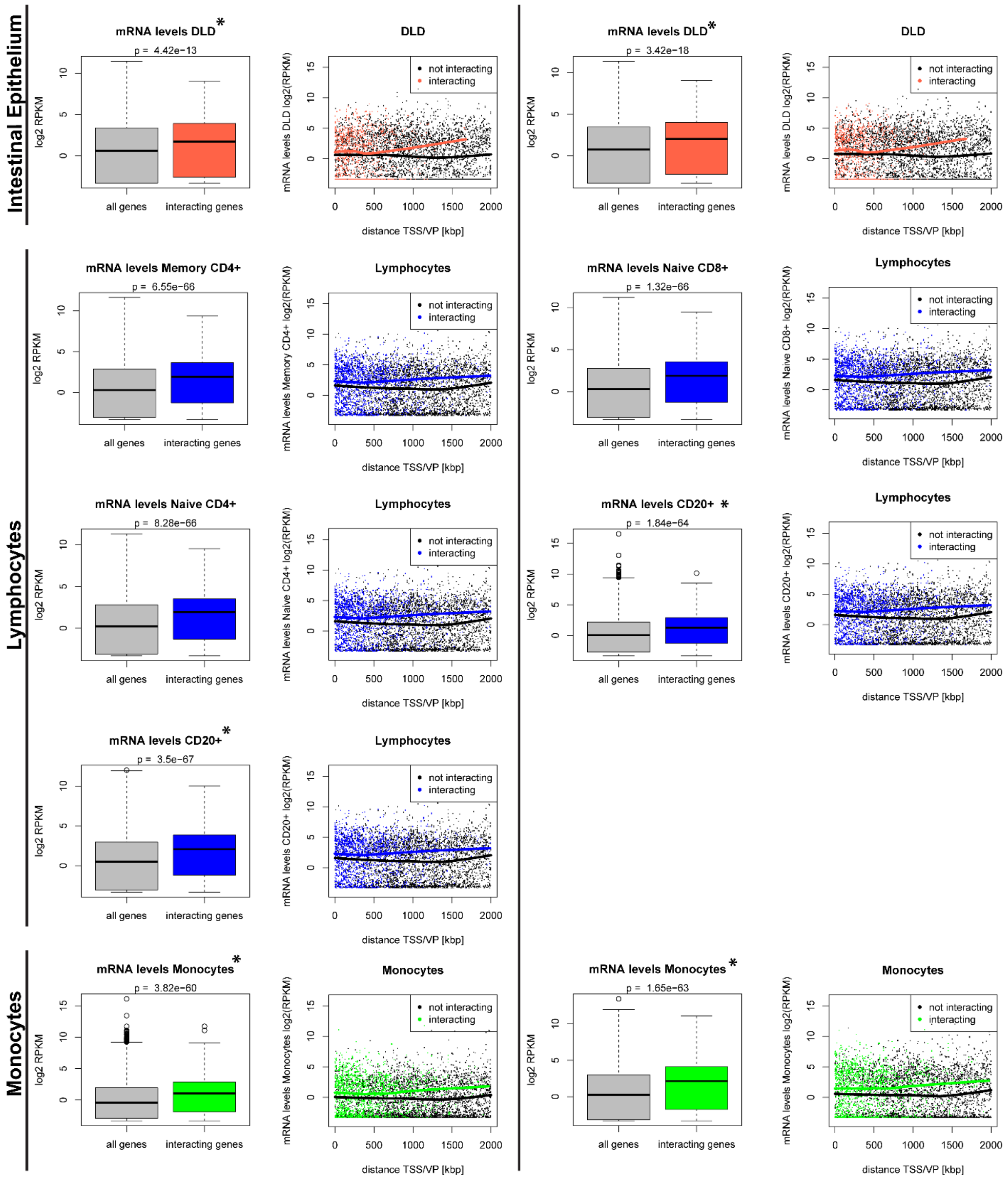

Supplementary figure 6. RNA expression. *Boxplots show that the mean RNA expression of regulated genes is higher than expression in all annotated genes. Each plot shows one RNA-seq data set. For all data sets, the genes interacting with the assayed regulatory elements are significantly higher expressed than all annotated genes. P-values are based on Mann-Whitney-U tests. The dot charts show expression levels of genes in the vicinity of the viewpoints. Genes that are interacting with the assayed enhancer are expressed at higher levels. *Biological duplicates for the same cell type.*

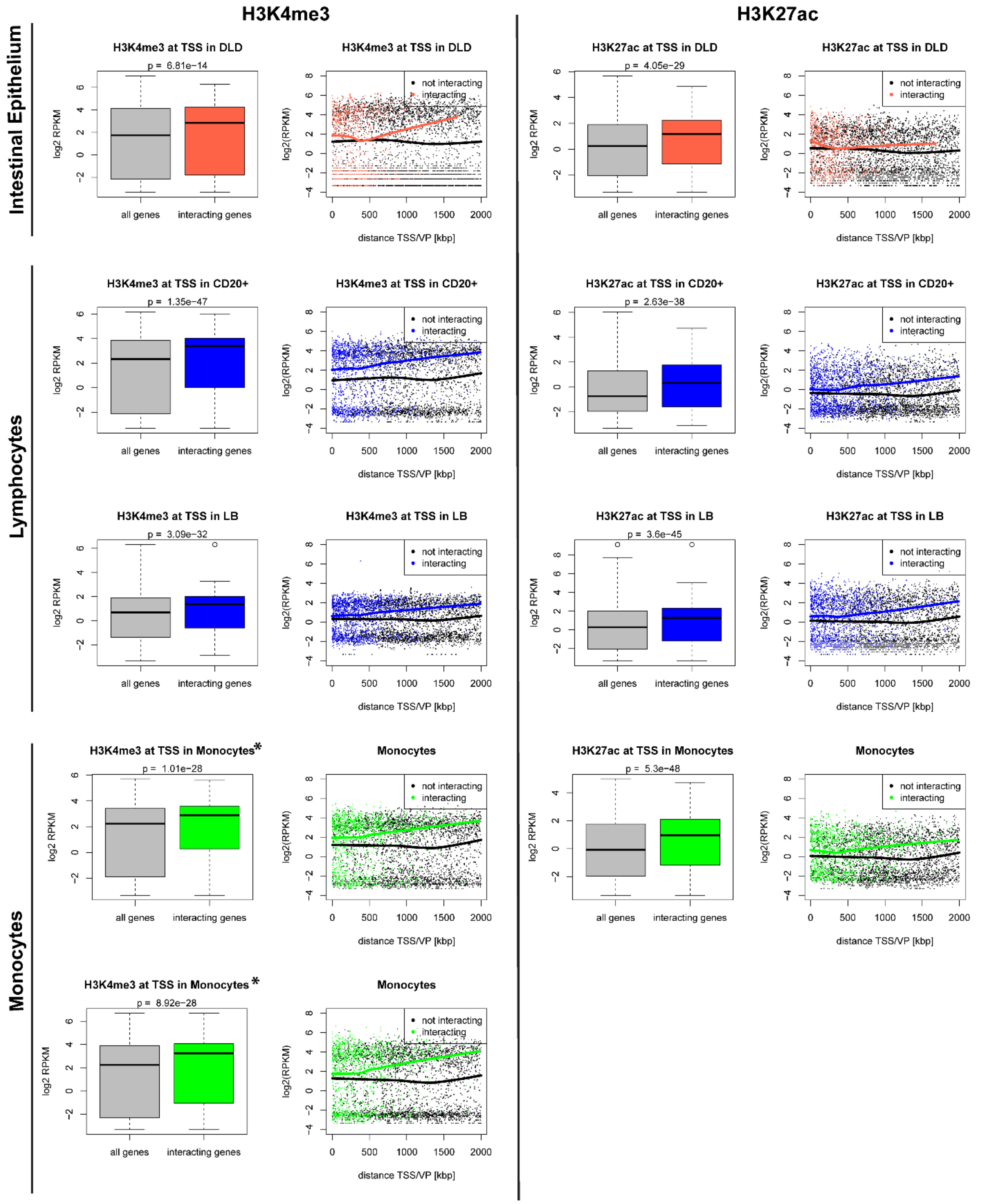

Supplementary figure 7. H3K4me3 and H3K27ac occupancy. *TSS occupancy by H3K4me3 and TSS occupancy by H3K27ac (right) in different cell types. Both H3K4me3 and H3K27ac occupancy is significantly higher at TSSs of genes detected with 4C-seq, p-values are based on Mann-Whitney-U tests. Dot plots depict TSS occupancy by H3K4Me3 and H3K27Ac in genes in the vicinity of the viewpoint (interacting and not interacting with the assayed enhancer). *Biological duplicates for the same cell type.*

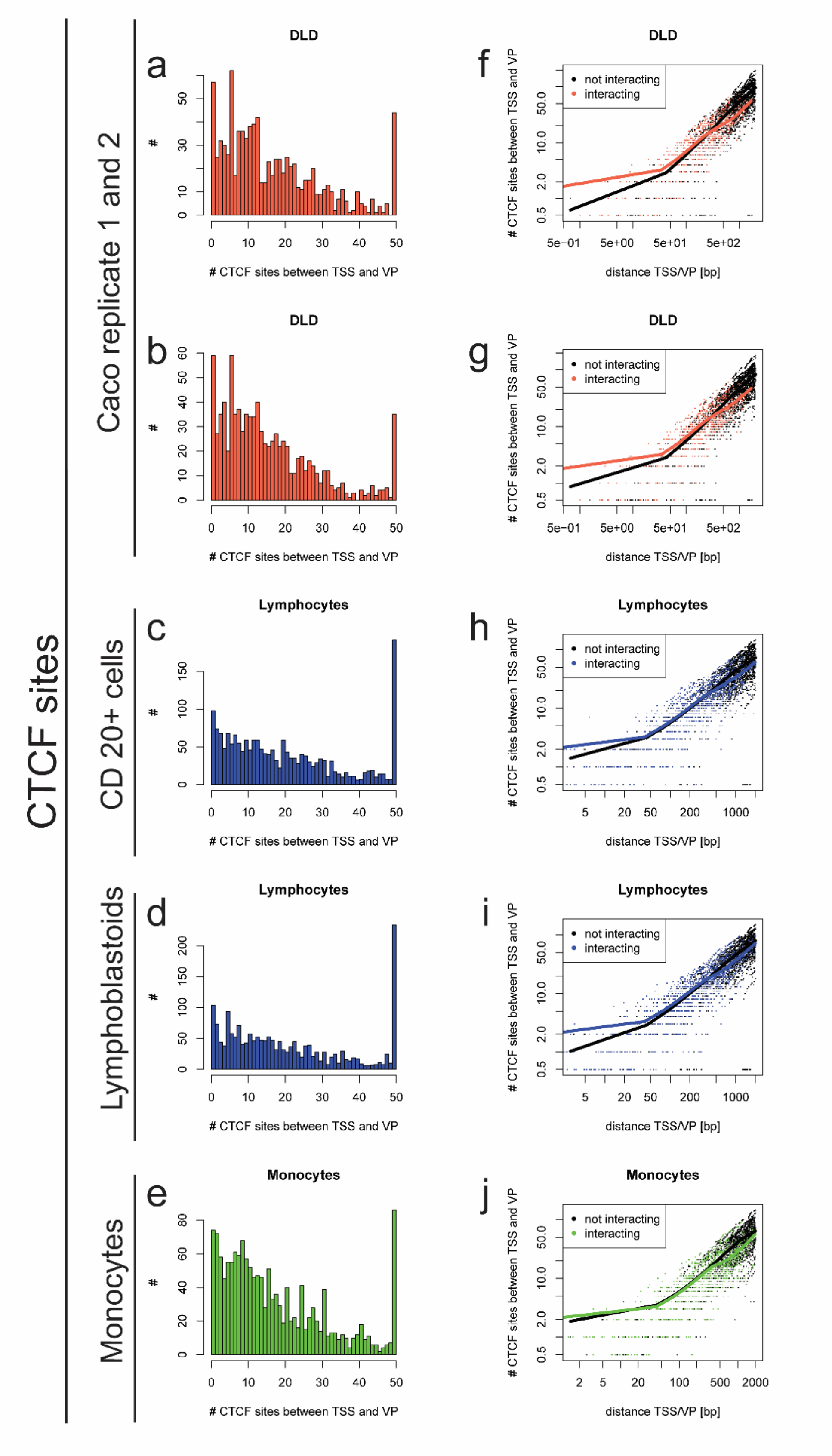

Supplementary figure 8. Insulators between viewpoint and candidate genes. a-e) Number of CTCF bound-loci between viewpoint and interacting gene. ***f-j)*** The number of CTCF occupied sites between a gene and the viewpoint does not differ for genes interacting with the viewpoint, compared to genes that do not interact with the viewpoint (i.e. genes that do not co-localize with significant 4C signal).

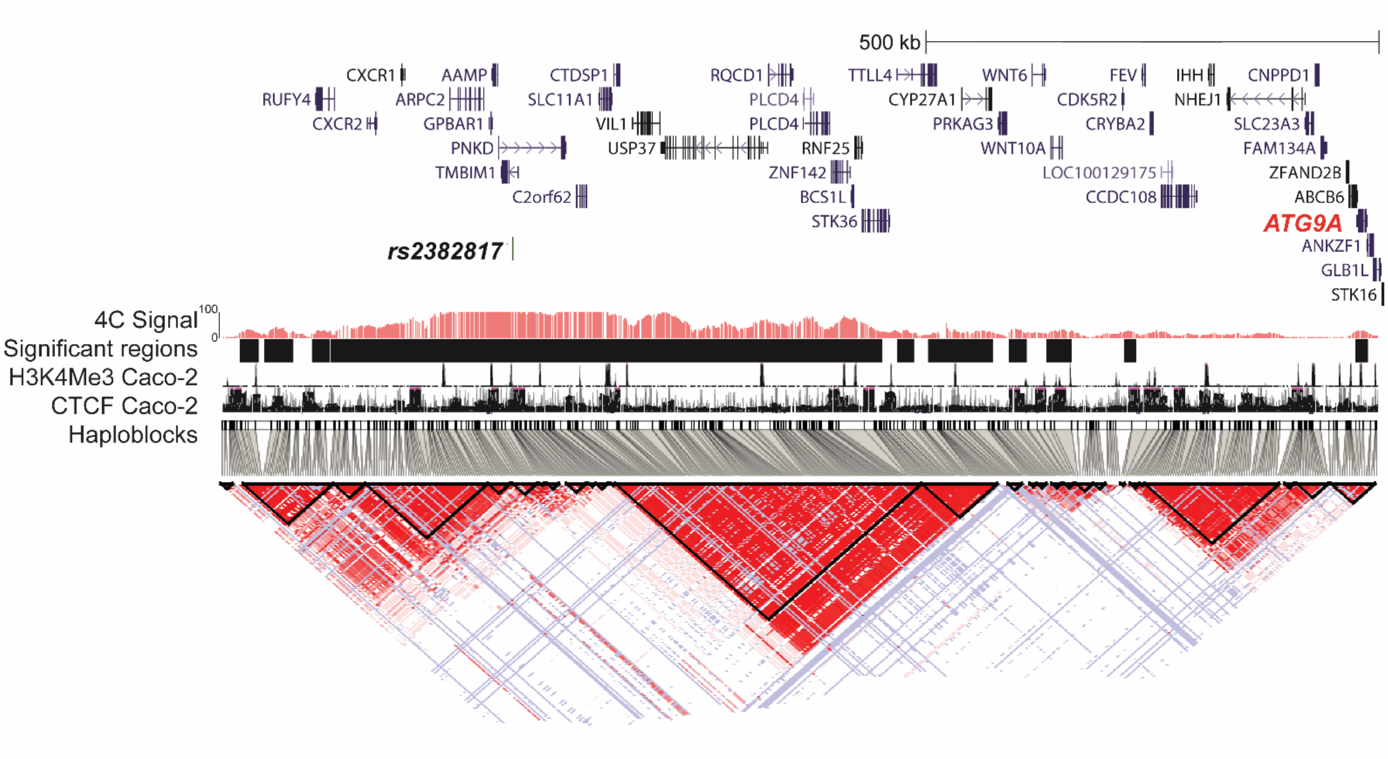

Supplementary figure 9. ATG9A is identified as a novel IBD candidate gene. *4C-seq results of rs2382817 in DLDs identifiy ATG9A as novel candidate gene. ATG9A localizes to a different haploblock then the IBD-associated SNP (rs2382817). 4C signal (red) of the rs2382817 locus is depicted on the y-axes as the percentage of fragends covered per pixel (see methods for details). Regions that are significantly enriched in 4C signal (P < 10^{-8}) are depicted by the black bars. ChIP-seq signals for H3K4Me3 and CTCF (in Caco-2 cell line, publicly available datasets from Encode consortium, see methods for additional information) and haploblocks (data retrieved from Haploview, see methods for additional information) are shown.*

SUPPLEMENTARY METHODS

Circular Chromatin Conformation Capture-Template preparation

4C-chromatin was prepared as described previously.[2] In brief, $10x10^6$ cells were used for chromatin preparation per cell type (monocytes, PBLs and DLD-1). Cells were crosslinked in 2% formaldehyde, lysed in lysis buffer and chromatin was isolated. Chromatin was digested with DpnII (NEB, #R0543L). After inactivation, the samples were diluted and ligated by T4 DNA ligase. Thereafter the second digestion was done using CviQI (NEB, #R069S) and inactivated by phenol:chloroform extraction. Finally, the chromatin was diluted, ligated and purified. Digestion and ligation quality were analyzed for the proper fragment lengths on agarose gels.

Primer design

Primer sequences are listed in *Supplementary table 1.* Primers were designed as was described previously.[2] In brief, primers were designed in a window of 5 kbp up- and downstream from the associated SNP. Forward and reverse primers were designed at least 300 bp apart. Forward (reading) primers were designed on top of the first restriction enzyme site. The reverse (non-reading) primer was designed close to (max 100 bp away from) the second restriction enzyme site. In case no primer pair could be designed within the initial window, the window was extended 5 kbp up- and downstream (n=22). If still no primer could be designed, we selected a primer pair that was less than 300 bp, but at least 240 bp, apart (n=2).

Circular Chromatin Conformation Capture- Sequencing (4C-seq) library preparation

4C-sequencing library preparation was performed as described previously,[2] with minor adaptations in order to make the protocol compatible with the large number of viewpoints: the PCR of 4C template was performed with 800 ng to 1,6 µg of 4C template per reaction. 4 to 10 primer pairs were multiplexed in the initial PCR reaction (primer sequences are listed in *Supplementary table 1*). Primers pairs were pooled according to primer efficiency. In reactions in which ≥6 primer pairs were used, PCR products were purified after an initial PCR reaction of 6 cycles (reaction volume = 200 µL) and divided among 8-10 PCR reactions containing single primer pairs for another 26 cycles (reaction volume = 25 µL). In PCR reactions in which <5 primer pairs were used, thermal cycling was limited to one reaction of 28 cycles.

Thereafter, PCR products derived from the same cells were pooled in equimolar amounts and a final 6 cycle PCR reaction containing 20 ng of pooled PCR product (reaction volume = 100 µL) was performed with primers that contained sequencing adaptor sequences (*Supplementary table 1*). All fragments >700 bp were removed using size selection on a 1% agarose gel follow by gel extraction of the selected products (Qiagen, #28704). Quality measures for the 4C library preparation and sequencing can be found in *Supplementary figure 1-3.*

eQTL analyses (STAGE)

The STAGE study was used to investigate the association between the identified GWAS loci and gene expression. The STAGE dataset consists of seven vascular and metabolic tissue samples of well-characterized coronary artery disease (CAD) patients gathered during coronary artery bypass grafting (CABG) as described[3]. Patients were included if they were eligible for CABG and had no other severe systemic diseases (e.g., widespread cancer and active systemic inflammatory disease). Fasting white blood cells were obtained for DNA and RNA isolation. The Ethical committee of the Karolinska Hospital approved this study and patients gave written consent (Dnr 004-02).

Genome-wide Human SNP array 6.0 (Affymetrix) was used for genotyping. From total 909,622 SNPs, 530,222 autosomal SNP passed quality control filters (minor allele frequency MAF < 5%, Hardy-Weinberg equilibrium (HWE) P<1e-6, and call rate of 100%). A custom-made HuRSTA-2a520709 was used for gene expression profiling from 109 genotyped patients (WB, n=102). A total 19,610 gene expression profiles were obtained. The missing autosomal SNP in STAGE study were imputed using IMPUTE2[4] after pre-phasing with SHAPEIT2[4] using 1000 Genomes (phase 1, version 3)[5]. A total of 5,473,585 autosomal SNPs were selected after filtering out low quality imputed genotypes (INFO score < 0.3).

Identified loci from GWAS for IBD were matched with imputed and genotyped SNPs and were selected for eQTL discovery. We used Matrix-eQTL[6] for investigating association between gene expression and SNPs. We compared the amount eQTLs between 'SNP-candidate gene'-pairs and 'SNP-control gene'-pairs. Control genes are genes within the same locus that are not interacting with the IBD associated locus. An empirical FDR was estimated for each eQTL-gene by shuffling patient IDs 1000 times on genotype data as described previously[7].

REFERENCES

1. Mifsud, B. *et al.* Sup Mapping long-range promoter contacts in human cells with high-resolution capture Hi-C. *Nat. Genet.* **47,** 598–606 (2015).

2. van de Werken, H. J. G. *et al. 4C technology: protocols and data analysis. Methods in enzymology* **513,** (Elsevier Inc., 2012).

3. Hägg, S. *et al.* Multi-organ expression profiling uncovers a gene module in coronary artery disease involving transendothelial migration of leukocytes and LIM domain binding 2: the Stockholm Atherosclerosis Gene Expression (STAGE) study. *PLoS Genet.* **5,** (2009).

4. Howie, B., Fuchsberger, C., Stephens, M., Marchini, J. & Abecasis, G. R. Fast and accurate genotype imputation in genome-wide association studies through pre-phasing. *Nat. Genet.* **44,** 955–9 (2012).

5. Abecasis, G. R. *et al.* An integrated map of genetic variation from 1,092 human genomes. *Nature* **491,** 56–65 (2012).

6. Shabalin, A. A. Matrix eQTL: ultra fast eQTL analysis via large matrix operations. *Bioinformatics* **28,** 1353–8 (2012).

7. Foroughi Asl, H. *et al.* Expression quantitative trait Loci acting across multiple tissues are enriched in inherited risk for coronary artery disease. *Circ. Cardiovasc. Genet.* **8,** 305–15 (2015).
